# Comparisons of sampling methods for assessing intra- and inter-accession genetic diversity in three rice species using genotyping by sequencing

**DOI:** 10.1038/s41598-020-70842-0

**Published:** 2020-08-19

**Authors:** Arnaud Comlan Gouda, Marie Noelle Ndjiondjop, Gustave L. Djedatin, Marilyn L. Warburton, Alphonse Goungoulou, Sèdjro Bienvenu Kpeki, Amidou N’Diaye, Kassa Semagn

**Affiliations:** 1 Africa Rice Center (AfricaRice), M’bé Research Station, 01 B.P. 2551, Bouaké, Côte d’Ivoire; 2 Université Nationale Des Sciences, Technologies, Ingénierie Et Mathématiques (UNSTIM), Abomey, Benin; 3 Corn Host Plant Resistance Research Unit, United States Department of Agriculture-Agricultural Research Service, Mississippi State, USA; 4 Crop Development Centre and Department of Plant Sciences, University of Saskatchewan, 51 Campus Drive, Saskatoon, SK S7N 5A8 Canada

**Keywords:** DNA sequencing, Next-generation sequencing, Natural variation in plants, Agricultural genetics, Population genetics

## Abstract

To minimize the cost of sample preparation and genotyping, most genebank genomics studies in self-pollinating species are conducted on a single individual to represent an accession, which may be heterogeneous with larger than expected intra-accession genetic variation. Here, we compared various population genetics parameters among six DNA (leaf) sampling methods on 90 accessions representing a wild species (*O. barthii*), cultivated and landraces (*O. glaberrima*, *O. sativa*), and improved varieties derived through interspecific hybridizations. A total of 1,527 DNA samples were genotyped with 46,818 polymorphic single nucleotide polymorphisms (SNPs) using DArTseq. Various statistical analyses were performed on eleven datasets corresponding to 5 plants per accession individually and in a bulk (two sets), 10 plants individually and in a bulk (two sets), all 15 plants individually (one set), and a randomly sampled individual repeated six times (six sets). Overall, we arrived at broadly similar conclusions across 11 datasets in terms of SNP polymorphism, heterozygosity/heterogeneity, diversity indices, concordance among genetic dissimilarity matrices, population structure, and genetic differentiation; there were, however, a few discrepancies between some pairs of datasets. Detailed results of each sampling method, the concordance in their outputs, and the technical and cost implications of each method were discussed.

## Introduction

The levels and distributions of intra-accession (within-accession) genetic diversity in genebank collections provide invaluable information for diverse purposes, including (a) deciding the number of seeds (plants) per panicle (ear) and the number of panicles per accession (or variety) that should be sampled and conserved to capture given attributes; and (b) serving as baseline data for germplasm management and distribution as well as monitoring genetic variation and integrity during conservation and regeneration^1–4^. Using limited numbers of accessions and/or agro-morphological traits and markers in different species, previous studies assessed intra-accession genetic diversity using morphological and isozymes^5^, amplified fragment length polymorphisms (AFLP)^3,4,6^, random amplified polymorphic DNA (RAPD)^7^, inter simple sequence repeat (ISSR)^8,9^, and simple sequence repeats (SSR) markers^10^. RAPD, AFLP, and ISSR markers are currently becoming obsolete for germplasm characterization for multiple reasons, including dominant inheritance, low reproducibility, low throughput for genotyping thousands of collections conserved at most genebanks, low marker density (genome coverage), poor resolution associated with the size-based fragment analysis system, and difficulty in merging multiple datasets generated by different collaborators or labs^11^. SSR markers are codominant and more reproducible, with better genome coverage than AFLP, RAPD and ISSRs; however, they are not well suited for large-sale characterization of genebank collections, primarily due to lower throughput, high genotyping cost, and difficulty in merging genotypic data generated by multiple collaborators or labs due to their ability in detecting multiple alleles, stuttering, and addition or omission of a nucleotide during polymerase chain reaction (± A) that causes ambiguity in automated fragment analysis systems using capillary DNA sequencers^12–14^.

The availability of low-cost next-generation sequencing (NGS) technologies that generate high-density genome-wide SNPs is providing genetic resource scientists tremendous opportunities to enhance the quality, efficiency, and cost-effectiveness of genebank operations^15,16^. These include germplasm curation^17^; generation of high-density reference genotypic data^18^ and molecular passport data^19^; gene discovery using genomewide association studies and selective sweep analysis^18–22^; understanding the genetic profiles of the entire collection^19,23^; identifying redundant collections and creating subsets of genetically unique accessions for genetic and breeding studies^19,24,25^; and correcting mislabeled, taxonomically misclassified and/or misidentified collections^26,27^. Using GBS, for example, nearly 33% of the 22,626 barley accessions at the Leibniz Institute of Plant Genetics and Crop Plant Research’s (Gatersleben, Germany)^19^ and 50% of the 1,143 accessions of a wild relative of wheat (*Aegilops tauschii*)^17^ were found to be potential duplicates.

Recently, our team at the AfricaRice center implemented a pilot study to characterize 4,115 rice accessions representing *Oryza barthii* A. Chev., *O. glaberrima* Steud. (African rice) and *O. sativa* L. (Asian rice) using DArTseq technology^28^. The DArTseq-based SNPs were highly useful for a wide range of purposes, including (1) understanding the genetic diversity, population structure, and genetic differentiation among African rice (*Oryza glaberrima* Steud.) collections, and developing core and minicore sets^25^; (2) developing species- and subspecies-diagnostic SNP markers to minimize misclassification, misidentification and mislabeling errors during germplasm acquisition and routine genebank operations^26^; (3) identifying candidate genes using selective sweep analysis^21^; and (4) comparing the extent of genetic variation and relatedness among various landraces and improved intraspecific and interspecific rice varieties developed by AfricaRice breeders with those developed by other institutions^29^. Based on the pilot study, we aim to genotype the entire rice collection conserved at AfricaRice using DArTseq and use the data to improve our germplasm curation. We will create subsets of the most genetically diverse accessions for further field evaluation, gene discovery, trait donor selection, and pre-breeding, which will ultimately promote the use of the collections in rice improvement. To reduce genotyping costs per accession, most molecular characterization studies in self-pollinating species are conducted by randomly sampling a single plant to represent an accession. This has been the case in our previous studies and other studies in rice^25,30,31^, barley^19^, and wild relatives of wheat^17^. Single plant samples have provided invaluable data for assessing inter-accession genetic diversity, relatedness and population structure in self-pollinating species, but are not suitable for measuring intra-accession diversity, which forms one of the bases of the current study. Furthermore, a single plant genotype data may be misleading when the extent of intra-accession diversity is greater than expected for different reasons, including a higher level of outcrossing^32,33^, phenotypic heterogeneity, seed admixture, pollen contamination and off-types, which is another basis for this study. For example, sorghum landraces and wild rice showed an outcrossing rates that varied from 5 to 40%^32^ and from 4 to 25%^33^, respectively. As a result, there is concern among the genetic resources scientists that results based on a single individual genotype may not be comparable with multiple plants per accession, genotyped either individually or in bulks^17^.

Bulk segregant analysis^34,35^ refers to the genotyping of bulks of individuals using either plant tissue bulking or DNA pooling^36^. In outcrossing species, the bulking method has been commonly used for quick and economic genotyping of inbred lines, populations, and open-pollinated varieties for different purposes^37–39^. In selfing species, however, bulk segregant analysis has been used primarily for mapping genes and quantitative trait loci (QTL) associated with target traits of importance in breeding^34,40–44^. Some researchers have recommended bulking (pooling) method for characterizing multiple individuals per accession as the basis for evaluating genetic identity and diversity within accession in self-pollinating species^15,17^, but this method also has its limitations, including knowing the minimum number of individuals required in the bulk^15^, and the sensitivity of the genotyping platforms in detecting rare alleles due to allele dilution problems^38,45^. The alternative method of genotyping multiple plants per accession individually may be ideal for capturing rare alleles and estimating intra-accession genetic diversity but will increase the genotyping costs per accession multi-fold. The objectives of this study were, therefore, to: (1) assess intra-accession and inter-accession genetic diversity in 90 rice accessions, each represented by six leaf sampling methods (a randomly selected single plant, 5 plants, 10 plants and 15 plants, bulks of 5 plants and bulks of 10 plants); (2) compare the concordance among the different sampling methods with respect to species (*O. barthii*, *O. glaberrima*, and *O. sativa*) and genetic backgrounds of the germplasm (wild vs. landraces vs. improved); and (3) compare the outputs of the different datasets and assess if there were cases where one method provided obvious advantages over the others as well as the cost and technical implications of each method for large-scale germplasm curation and characterization in selfing species.

## Methods

### Plant materials and genotyping

**Figure 1:**
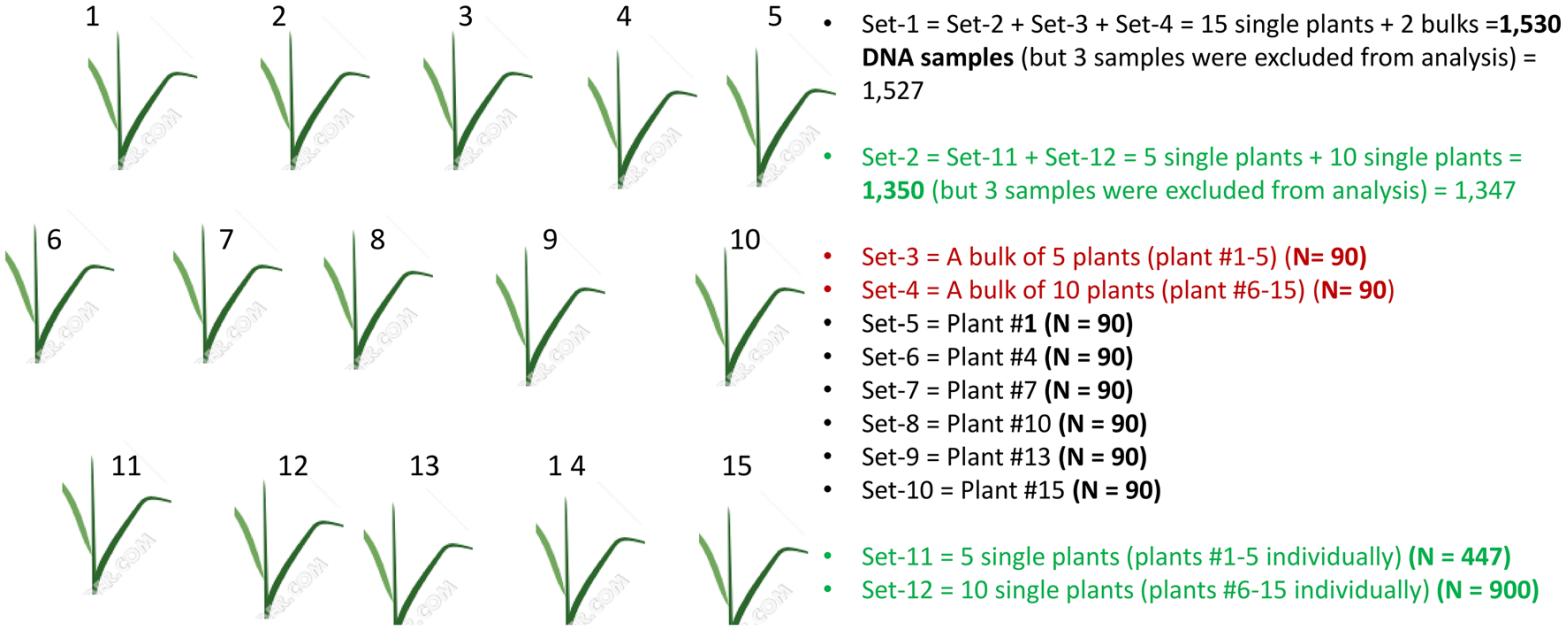
Outline of the DNA (leaf) sampling methods used in each of the 90 accessions. Each accession was originally represented by 15 individuals (plant numbered from 1 to 15), a bulk of 5 plants (plant #1–5), and another bulk of 10 plants (plant #6–15).

This study was conducted using a total of 1,527 DNA samples from 90 accessions and varieties (all referred here as accessions) that represented a wild *O. barthii* (18), landraces of cultivated species of *O. glaberrima* (21), *O. sativa* subsp. indica (19), *O. sativa* subsp. japonica (18), and improved interspecific varieties/genotypes derived from crosses between *O. glaberrima* and *O. sativa* (14) (Supplementary Table S1). The 90 accessions were part of the rice germplasm used in our previous studies for the development of species- and subspecies-diagnostic SNP markers^26^ and for comparing diversity indices and selective sweeps^21^. Each accession was represented by 17 DNA samples (Fig. 1) comprised of 15 single plants, a bulk of 5 plants (plants numbered 1–5), and another bulk of 10 plants (plants numbered 6–15). The detailed procedures for genomic DNA extraction and SNP genotyping using DArTseq have been described in our previous study^25^. Each DNA sample was genotyped with 67,728 SNPs by the DArT Pty Ltd, Australia (https://www.diversityarrays.com). Three DNA samples had over 70% missing data points and were excluded from the dataset. The genotype data of the remaining 1,527 samples were imputed using Random Forest^46^, which is implemented as “randomForest” in the R package^47^.

### Statistical analyses

To evaluate the accuracy of the imputed SNPs in genetic diversity and population structure analyses, we first computed identity-by-state (IBS)-based genetic distance matrices from the 67,728 SNPs before and after imputation and compared the two distance matrices using the Mantel test^48^ implemented in NTSYSpc v2.1^49^. Because genotyping errors may account for about 1% of observed differences^26,50,51^, it is often difficult to consider SNPs with minor allele frequency < 0.01 as polymorphic sites. For that reason, we filtered the imputed genotype data using a minor allele frequency (MAF) of 0.01 and maximum heterozygosity of 0.50, which formed dataset Set-1 that consisted of 15 individual samples and two bulks. In this study, we used heterozygosity for simplicity to refer both to heterozygosity in the individuals (single plants) and heterogeneity in the bulks. Eleven additional subsets of data were created from Set-1 corresponding to all 15 plants individually (Set-2), a bulk of 5 plants (Set 3), another bulk of 10 plants (Set-4), and randomly selected individuals from Set-2 repeated 6-times (Set-5 to Set-10), 5 plants individually (Set 11) and 10 plants individually (Set 12).

Most of the statistical analyses were performed as described in previous studies^20,25^. Briefly, heterozygosity, IBS-based genetic distance matrices, and principal component analysis (PCA) were computed using TASSEL v.5.2.58^52^. The first two principal components (PCs) from the PCA were plotted for visual examination in XLSTAT 2012 (Addinsof, New York, USA; www.xlstat.com) using species/subspecies and predicted group memberships from phylogenetic and population structure analyses as categorical variables. The correlation between pairs of genetic distance matrices was computed using the Mantel test^48^ implemented in NTSYSpc v2.1^49^. The HapMap format of each dataset was exported to PHYLIP interleaved format using TASSEL v.5.2.57, which was then converted to MEGA X^53^, STRUCTURE v.2.3.4^54^ and ARLEQUIN v.3.5.2.2^55^ formats using PGDSpider v.2.1.1.3^56^. We used Molecular Evolutionary Genetics Analysis (MEGA) X to compute the pairwise maximum composite likelihood (MCL)-based genetic distance between DNA samples and accessions, for constructing phylogenetic trees using the neighbor-joining method, and for computing number of segregating sites (S), the proportion of polymorphic sites (Ps), Theta (θ), and nucleotide diversity (π). A site (SNP) was considered segregating if it had two or more nucleotides at that site; π refers to the average number of pairwise nucleotide differences between two sequences (samples), while θ was used as another estimator of diversity parameters based on the number of segregating sites in the samples. Phylo.io^57^ was used for comparing pairs of phylogenetic trees side-by-side as well as for computing Robinson-Foulds (RF) distance^58^ and number of subtree prune-and-regraft (SPR) distances^59,60^ between pairs of phylogenetic trees. For such purposes, Newick files were generated for each dataset using MEGA X and used as inputs into Phylo.io.

Population structure was analyzed using the model-based method implemented in the software package STRUCTURE v.2.3.4^54^ as described in the previous studies^20,25,61^. DNA samples and accessions with membership probabilities > 60% were assigned to the same clusters (group), while those with probabilities < 60% in any group were assigned to a “mixed” group. Analysis of molecular variance (AMOVA)^62^ and F_ST_-based pairwise genetic distance matrices^63^ were computed among and within groups using ARLEQUIN v.3.5.2.2^55^. Accessions were assigned into 3–5 groups (populations) based on their species/subspecies, ecologies or group membership predicted from the phylogenetic and population structure analyses.

## Results

### Intra-accession diversity

Of the 67,728 SNPs used for genotyping the 1,527 DNA samples (Supplementary Table S2), the proportion of missing data per SNP and sample before imputation varied from 0 to 64.1% for single plants and from 4.2 to 61.1% for bulks, with an overall average of 20.8%. In the initial genotyping data set, 69.1% of the markers (46,818 SNPs) were polymorphic across the 1,527 samples (Set-1), each with a minor allele frequency varying from 0.01 to 0.050 (Supplementary Table S3). Pearson correlation coefficients between minor allele frequency and heterozygosity estimated before and after imputation were high, at 0.983 and 0.998, respectively. The Mantel test performed on genetic distance matrices computed from all SNPs before and after imputation also revealed a very high positive correlation (r = 0.987). Hence, detailed results are presented only for the imputed version of the 46,818 polymorphic SNPs.

**Figure 2:**
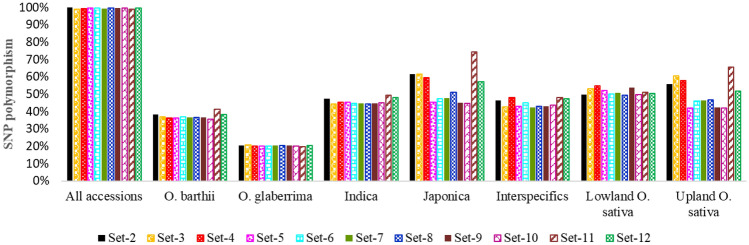
Summary of the percentages of polymorphic SNPs used for statistical analyses of all accessions (N = 90), *O. barthii* (18), *O. glaberrima* (21), *O. sativa* subsp. indica (19), *O. sativa* subsp. japonica (18), improved interspecific genotypes (14), lowland *O. sativa* (30), and upland *O. sativa* (21). See Supplementary Table S1 for germplasm summary and Table S2 for details on the number of polymorphic SNPs for all datasets.

We assessed intra-accession diversity from Set-2, Set-11, and Set-12 that consisted of genotypic data of 15, 5, and 10 individuals, respectively. The percentage of SNP polymorphism, allele frequencies, heterozygosity, θ, π, and genetic distance between pairs of individuals belonging to the same accession are used as indicators of intra-accession genetic diversity. The level of SNP polymorphism across the 90 accessions was highly similar across the different datasets (Fig. 2), which was 99.5–99.7% for single plants, 98.8–99.9% in the 5–15 individual plants, 98.9–99.2% in the bulks (Table 1, Supplementary Table S2). Observed heterozygosity per accession computed from 5, 10 and 15 DNA samples ranged from 0.5 to 25.7, from 0.2 to 12.3% and from 0.2 to 25.7%, respectively (Supplementary Table S1, Fig. S1). Only 11 accessions had observed heterozygosity exceeding 6% for at least one individual (three accessions in all Set-2, Set-11, and Set-12; four accessions in both Set-2 and Set-11; four accessions in both Set-2 and Set-12), which is the expected average outcrossing rate reported in cultivated rice^30,64^. The average heterozygosity per accession estimated from all sets of 5, 10 and 15 individuals ranged from 0.5 to 5.6%, from 0.5 to 4.0% and from 0.5 to 3.8%, respectively (Supplementary Table S1).

**Table 1:** Summary of polymorphic SNPs selected for statistical analyses of 90 accessions in all datasets.

Dataset	No. of plants per accession	Total sample size	No. of polymorphic SNPs	Proportion of SNP polymorphism as compared to Set-1 (%)
Set-1	15 individuals and 2 bulks	1527	46,818	100.0
Set-2	15 individuals	1347	46,774	99.9
Set-3	A bulk of 5 plants	90	46,322	98.9
Set-4	A bulk of 10 plants	90	46,446	99.2
Set-5	Single plant	90	46,601	99.5
Set-6	Single plant	90	46,678	99.7
Set-7	Single plant	90	46,573	99.5
Set-8	Single plant	90	46,649	99.6
Set-9	Single plant	90	46,647	99.6
Set-10	Single plant	90	46,595	99.5
Set-11	5 individuals	447	46,276	98.8
Set-12	10 individuals	900	46,716	99.8

**Figure 3:**
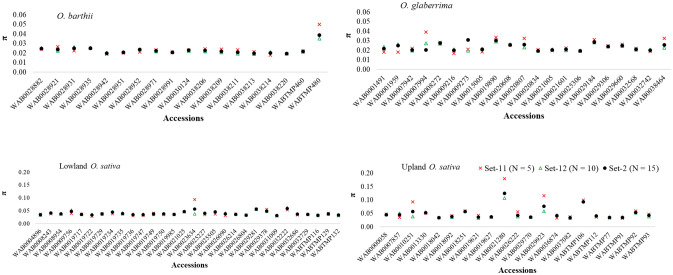
Summary of nucleotide diversity (π) computed as measures of intra-accession genetic diversity in *O. barthii* (18), *O. glaberrima* (21), lowland *O. sativa* (30) and upland *O. sativa* (21). Each accession was represented by 15 single plant DNA samples genotyped with 48,818 SNPs. See Supplementary Table S1 for germplasm summary and Table S4 for molecular diversity indices of each accession.

**Figure 4:**
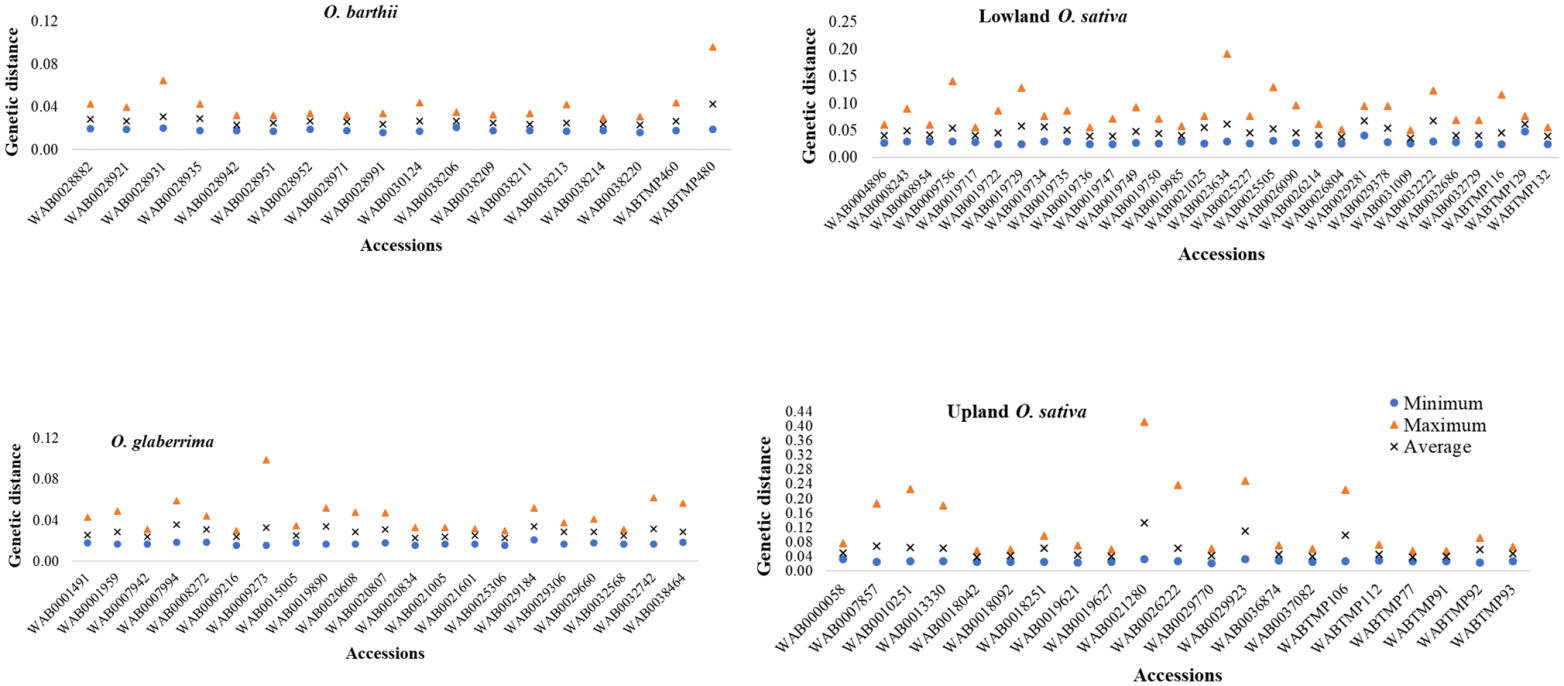
Comparisons of minimum, maximum, and average genetic distance values computed between pairs of 15 individuals sampled per accession in Set-2, each genotyped with 48,818 SNPs. See Supplementary Table S8 for details.

**Figure 5:**
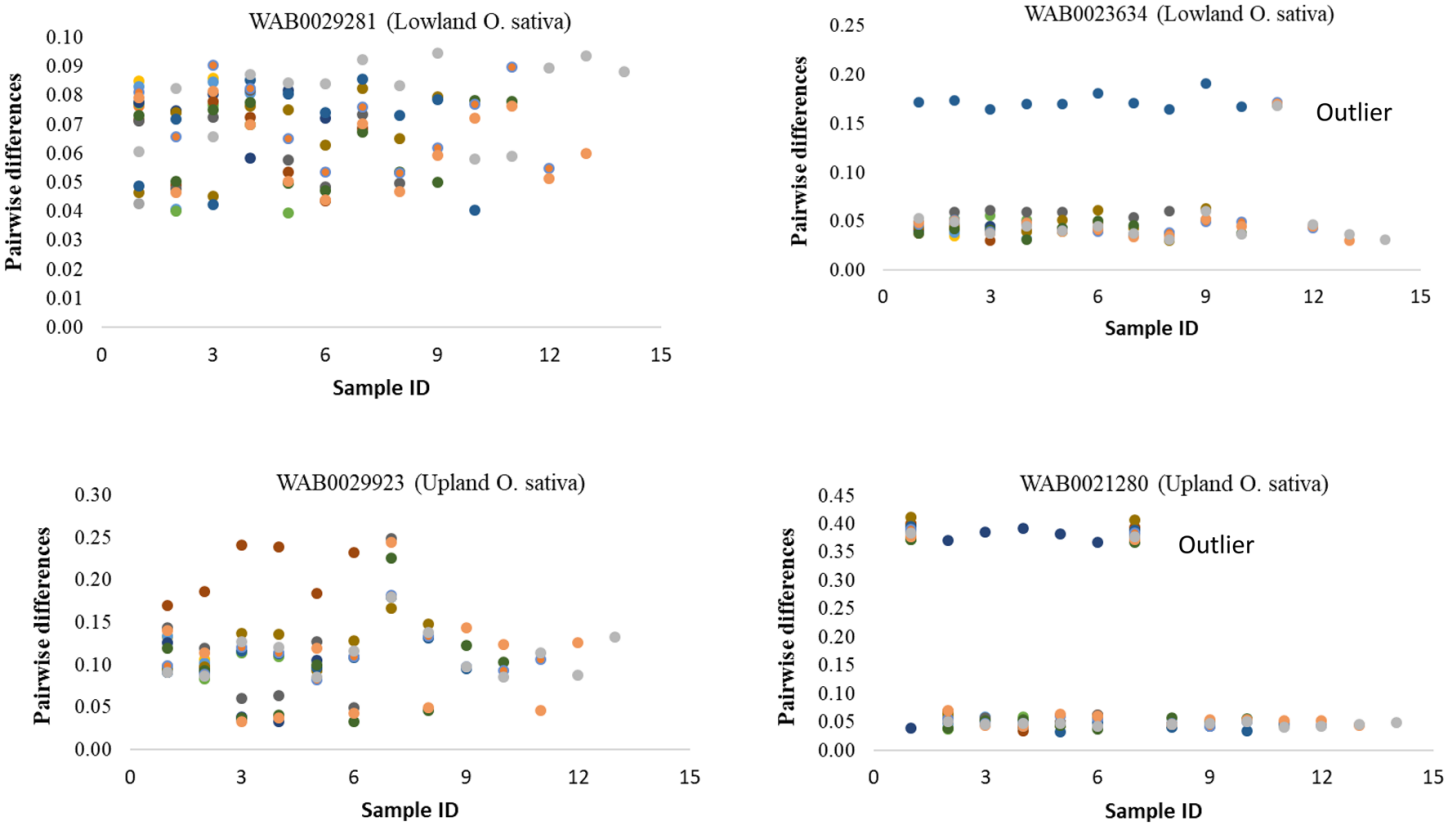
A plot of identity-by-state-based genetic distance values computed within 4 accessions, each represented by 15 single plant DNA samples genotyped with 48,818 SNPs. Genetic distances between pairs of individuals within WAB0029281 and WAB0029923 were within the expected range for self-pollinated species, while WAB0023634 and WAB0021280 have outlier individuals. See Supplementary Figure S3 for 10 more accessions that had larger than expected intra-accession diversity.

As summarized in Fig. 3 and Supplementary Table S4, θ and π computed within every accession ranged from 0.017 to 0.205 based on 5 plants per accession; from 0.019 to 0.149 based on 10 plants, and 0.019–0.140 based on 15 plants, which is an indication of a relatively low intra-accession diversity and more homogenous seed lot within most accessions. Values for θ and π estimated from Set-2, Set-11 and Set-12 within 90 accessions were highly correlated (0.967 ≤ r ≤ 0.996) and very low, with 81 of the 90 accessions showing < 0.06 θ and π values (Supplementary Fig. S2, Supplementary Table S4). However, nine *O. sativa* accessions adapted to the lowland (WAB0009756, WAB0023634, and WAB0032222) and upland (WAB0007857, WAB0010251, WAB0013330, WAB0021280, WAB0029923, WABTMP106) ecologies had θ and/or π values ranging from 0.061 to 0.205 in at least one of the three datasets, which may be due to broader intra-accession diversity or to errors that might have occurred during genotyping and/or sample preparation (e.g., seed mix up during planting, labeling error, contamination during leaf sampling or DNA extraction). To determine the cause of such unexpectedly large intra-accession diversity within these accessions, we compared pairwise IBS-based genetic distance for the 15 individuals in Set-2. Figure 4 and Supplementary Table S1 summarizes the minimum, maximum, and average genetic distance between pairs of individuals within each accession. Pairs of individuals belonging to the same accession differed between 1.6 and 41.2% of the scored alleles, of which 48 accessions differed by ≤ 6% of the alleles of the 46,818 SNPs. The remaining 42 accessions showed at least a pair of individuals that differed by > 6% of the scored alleles, which is due to either greater intra-accession diversity or due to the presence of outliers. Figure 5 and Supplementary Fig. S3 demonstrates intra-accession diversity of some accessions with and without potential outliers.

**Figure 6:**
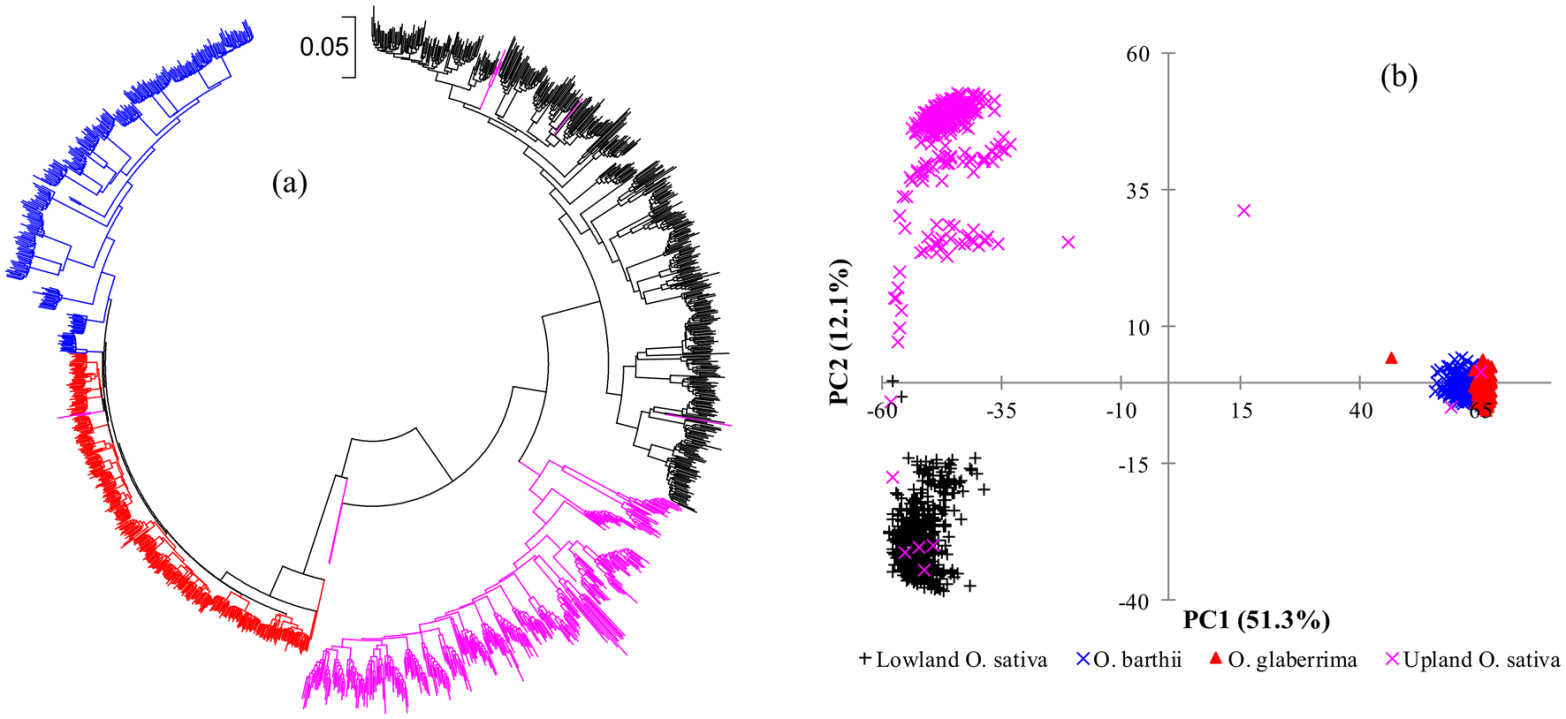
**(a)** Neighbor-joining tree constructed using Molecular Evolutionary Genetics Analysis (MEGA) X (https://www.megasoftware.net/), and **(b)** plots of PC1 and PC2 from principal component analyses of 1,347 single plant DNA samples in Set-2 based on 46,818 SNPs. In both figures, samples belonging to the same group have the same font color: *O. glaberrima* (red), *O. barthii* (blue), *O. sativa* adapted to the upland ecology (pink) and lowland ecology (black). See Supplementary Table S1 for group membership and Supplementary Table S8 for genetic distance matrices.

Figure 6 shows a neighbor-joining phylogenetic tree and a principal component analysis plot of the 1,347 individuals in Set-2. In the phylogenetic tree, all individuals from 61 of the 90 accessions (67.8%) tend to be more similar to each other and clustered together as expected, while 25 accessions (27.8%) had 1–4 individuals that clustered with other accessions belonging to either the same or a different species/subspecies. Overall, 47 of the 1,347 individuals (3.5%) from 25 accessions were suspected outliers, which included *O. barthii* (2), *O. glaberrima* (12), *O. sativa* (29); the latter includes indica (2), japonica (15) and interspecific genotypes (12). The 15 individuals from each of 4 other accessions were divided into two distinct but genetically similar sub-clusters.

We observed nine accessions (WAB0023634, WAB0029281, WAB0032222, WAB0010251, WAB0013330, WAB0018251, WAB0021280, WAB0029923, and WABTMP106) that differed by at least 5% of the scored alleles based on π, θ and IBS-based genetic distance between pairs of individuals from within the accession; all these accessions except WAB0029281 each had 1–3 samples that did not cluster together with the other individuals originating from the same accession in the phylogenetic tree. The first five principal components from PCA performed in Set-2 accounted for 70.6% of the variation observed across the 1,347 individuals (Supplementary Table S5). A plot of PC1 (51.3%) and PC2 (12.1%) revealed a similar grouping pattern as the neighbor-joining analysis (Fig. 6). However, all individual samples originating from *O. barthii* and *O. glaberrima* appeared nearly identical in the PCA plot because only 40% of the 46,818 SNPs were polymorphic within these two species as compared to 65% of the SNPs that were polymorphic among the *O. sativa* accessions.

### Inter-accession diversity in multiple datasets

Using genotypic data of all accessions, we compared SNP polymorphisms, heterozygosity, θ, π, and genetic dissimilarity across the twelve datasets. Of the 48,818 SNPs that were polymorphic across the 1,527 single plants and bulked DNA samples in Set-1, 98.8 to 99.9% of the SNPs were polymorphic in the datasets represented by a randomly selected single plant, 5–15 single plants, and bulks of either 5 or 10 plants. Marker polymorphisms computed among accessions belonging to the four different species and eco-geographical groups demonstrated highly similar patterns of polymorphism irrespective of the DNA sampling methods and genetic background of the germplasm (Fig. 2). For example, the lowest (19.7–20.6%) marker polymorphism was observed within *O. glaberrima*, which was very consistent whether each accession was represented by a randomly selected individual, multiple individuals ranging from 5 to 15, or bulks. Pearson correlation coefficients between minor allele frequencies ranged from 0.993 to 1.00 (mean of 0.996) and heterozygosity estimated per SNP ranged from 0.902 to 1.00 (mean of 0.965) across all datasets (Supplementary Table S6). Observed heterozygosity per accession computed across all datasets ranged from 0.2 to 25.7% (Supplementary Table S1), with an overall average of 1.1%. Fourteen of the 90 accessions (15.6%) had an observed heterozygosity > 6.0% in one or more datasets (Supplementary Fig. S4), of which WAB0007857 and WAB0029923 were the most heterozygous accessions represented by 5 and 8 DNA samples with > 6.0% heterozygosity, respectively. Overall, approximately 84% of the 90 accessions had consistently < 6% heterozygosity across all datasets irrespective of the DNA sampling methods (Supplementary Fig. S4, Supplementary Table S1).

**Figure 7:**
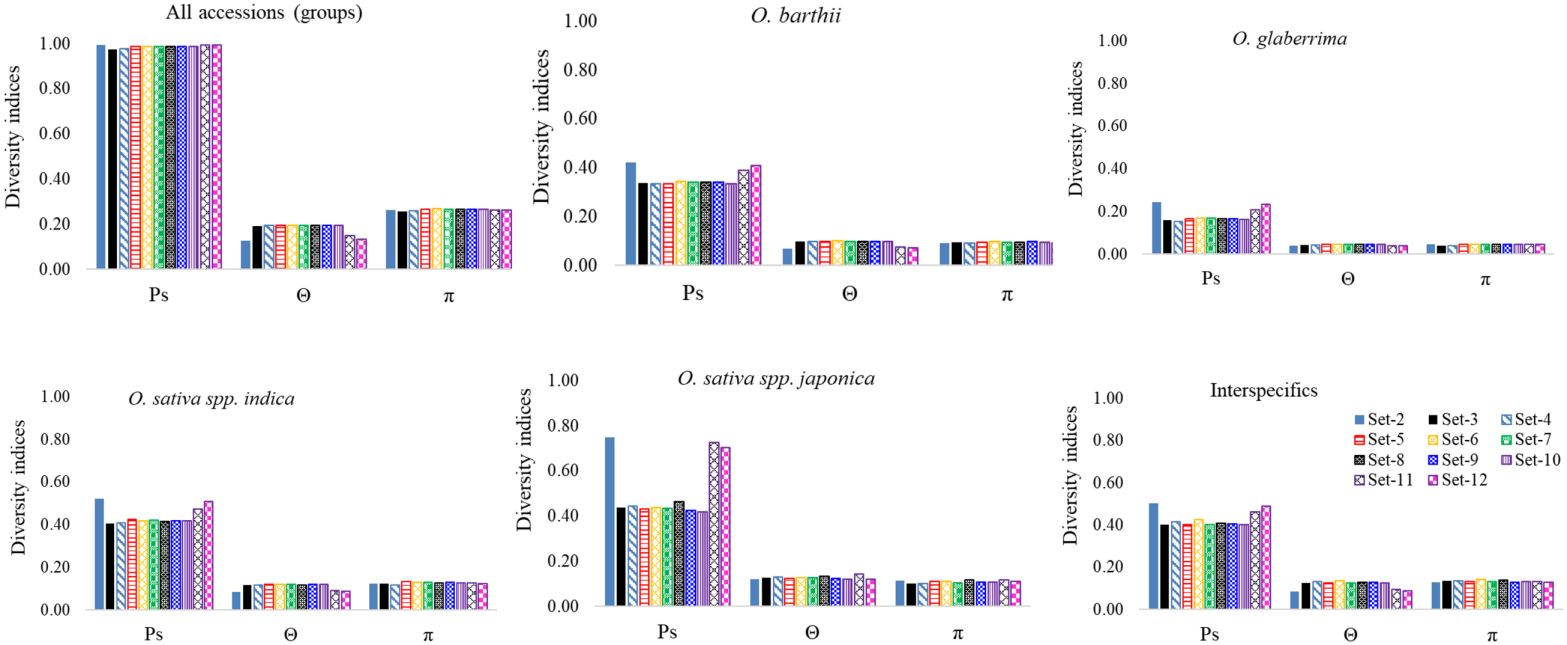
Summary of the proportion of polymorphic sites (Ps), θ and π across all datasets based on 48,818 SNPs. This figure was constructed using Microsoft Excel. See Supplementary Table S7 for details. Interspecific refers to improved genotypes derived from crosses between *O. glaberrima* and *O. sativa*.

We examined the overall genetic diversity indices across all datasets by assigning accessions into groups (Fig. 7, Supplementary Table S7). When all 90 accessions were used for analyses, Ps, θ and π estimated across all datasets varied from 0.976 to 0.995, from 0.128 to 0.195 and from 0.257 to 0.268, respectively, and each parameter was highly similar across datasets except relatively smaller values for θ when genotyping was done on 5–15 individuals in Set-2, Set-11, and Set-12. When we repeated the analyses using groups, Ps and θ values computed from Set-3 to Set-10 as well as π values estimated from all datasets showed similar patterns irrespective of the genetic background. On the other hand, Ps was larger and Θ was smaller when computed from the 5–15 individuals in Set-2, Set-11, and Set-12 compared to all other datasets. Overall, observed nucleotide diversity within *O. glaberrima* across all datasets, as measured by π, accounted for 40.9–50.1%, 33.4–51.9% and 28.5–35.9% of those of the wild *O. barthii*, the two *O. sativa* subspecies and the improved interspecific genotypes, respectively (Fig. 7, Supplementary Table S7).

### Genetic distance and population structure

**Figure 8:**
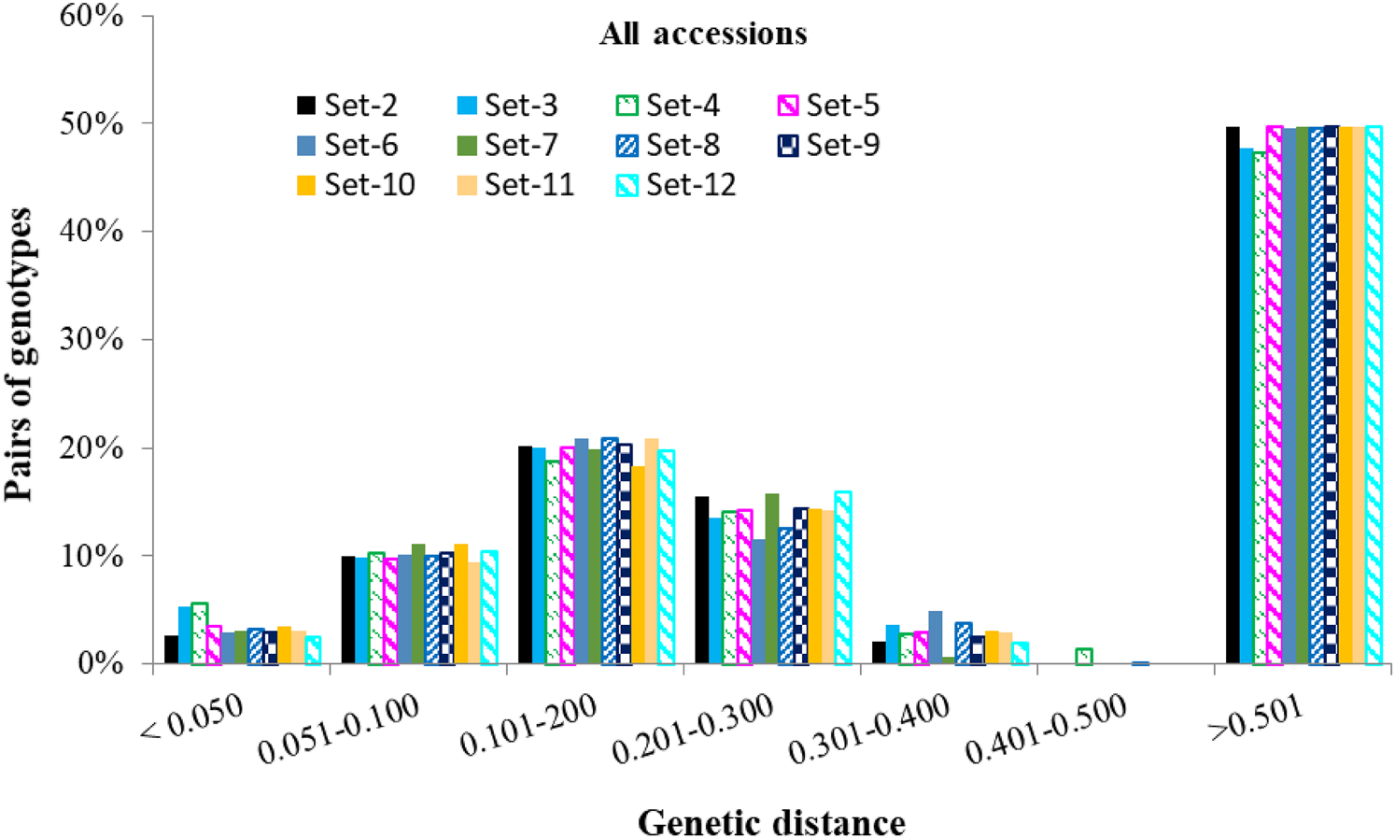
Frequency distribution categories of pairwise genetic distance between pairs of accessions computed from 11 datasets, each with 46,818 SNPs. See Supplementary Table S8 for details.

The genetic distance matrices computed between pair of the 90 accessions across all datasets are summarized in Fig. 8 and Supplementary Fig. S5. Overall, the minimum, maximum, and average pairwise genetic distances of the 90 accessions were highly similar irrespective of the DNA sampling methods. For example, the mean genetic distance between all pairs of the 90 accessions computed from the 5–15 single plants per accession as well as the bulk of five and ten plants varied from 0.019 to 0.697, from 0.021 to 0.732, and from 0.013 to 0.725, respectively. Mantel tests revealed a very high positive correlation among distance matrices between pairs of accessions (Supplementary Table S8) computed from all datasets, which ranged from 0.925 to 0.998 in all accessions (Supplementary Fig. S6). To determine if the genetic background of the germplasm influenced the correlations, we compared genetic distance matrices between pairs of accessions belonging to (a) *O. glaberrima*, *O. barthii*, *O. sativa* spp. indica, *O. sativa* spp. japonica and interspecific improved genotypes, and (b) the three groups predicted based on cluster analyses, PCA and the model-based population structure analyses (see below). Mantel correlations between datasets varied from 0.270 to 0.991 in *O. glaberrima*, from 0.878 to 0.999 in *O. barthii*, from 0.786 to 0.999 in indica, from 0.741 to 0.995 in japonica, and from 0.906 to 0.999 in interspecific improved genotypes. The lowest Mantel correlation coefficients were, therefore, observed within *O. glaberrima*, which is also evident from relatively inconsistent frequency distributions of the genetic distance matrices. When groups predicted based on the multivariate methods were considered, Mantel correlation coefficients among the distance matrices computed from all datasets were higher in the *O. glaberrima/O. barthii* group (0.945 ≤ r ≤ 1.000), followed by *O. sativa* adapted to the lowland (0.878 ≤ r ≤ 0.999) and upland (0.749 ≤ r ≤ 0.990) ecologies (Supplementary Table S9).

We examined the neighbor-joining tree constructed from the genetic distance matrix of all 1,527 samples in Set-1 to assess if the bulks of 5 and bulk of 10 plants consistently clustered with the 15 individual samples, which was observed among the 85 and 87 of the 90 accessions, respectively. About 96.4% of 1,527 single plants and bulked DNA samples originating from the same accession were clustered together as expected, while 3.5% of the individual and bulked samples from 26 accessions appeared to be potential outliers (Supplementary Fig. S7). DNA samples that were found to be potential outliers or mis-clustered are likely errors for different reasons, including admixture, contamination, and mislabeling during sampling, DNA extraction, and genotyping. We then assessed population structure among the 90 accessions to determine how they tended to cluster into groups across all datasets. Overall, the neighbor-joining tree constructed from the genetic distance matrix computed from Set-2 showed three major groups (Fig. 6). Accessions belonging to *O. glaberrima* and *O. barthii* formed the first group. In contrast to both *O. glaberrima* and *O. barthii* accessions that did not show any population structure by their ecology of origin, *O. sativa* accessions formed two separate groups that were consistent with their adaptation ecologies. All *O. sativa* accessions and interspecific genotypes that are adapted to the lowland (primarily indica) and the upland (primarily japonica) ecologies were assigned into the second and third groups, respectively. The phylogenetic trees constructed from the other datasets revealed similar grouping patterns and are summarized in Supplementary Fig. S7. Overall, accessions belonging to each species and/or subspecies consistently clustered together across all datasets irrespective of the leaf (DNA) sampling methods with two exceptions. In Set-2, Set-6, Set-7, Set-10, Set-11, and Set-12, an *O. barthii* accession (WAB0028942) clustered together with *O. glaberrima* accessions, while another *O. barthii* accession (WAB0038213) clustered with *O. glaberrima* in Set-2, Set-11, and Set-12. Robinson-Foulds (RF) and SPR distances computed as measures of differences (disagreements) between pairs of the neighbor-joining phylogenies constructed from all datasets varied from 0.15 to 0.76 (RF) and from 7 to 37 (SPR) (Supplementary Table S10). The highest agreement (with the lowest RF value of 0.15 and SPR value of 7) was observed between phylogenies constructed from the 15 individuals in Set-2 and 10 individuals in Set-12.

The model-based population structure analyses revealed three distinct groups, similar to the phylogenetic analysis, with an *O. barthii/O. glaberrima* group, and two *O. sativa* groups adapted to the lowland and upland ecologies (Supplementary Table S1). The first five principal components from PCA performed across all datasets accounted for 70.6 to 72.3% of the molecular variation (Supplementary Table S5). A plot of PC1 and PC2 from all datasets also showed three distinct groups similar to the model-based population structure and the neighbor-joining analyses (Fig. 6, Supplementary Fig. S8). The DNA samples that we considered as potential outliers in the phylogenetic trees were also evident in the PCA plots.

### Genetic differentiation

The partitioning of the molecular variances of the various datasets into three, four, and five groups using AMOVA revealed that differences in groups accounted from 70.8 to 73.0%, from 69.7 to 71.9%, and from 66.8 to 68.4%, of the total variation, respectively. From 27.0 to 33.2% of the genetic variation resided within accessions irrespective of the dataset (sampling methods) and the number of groups used in the analyses (Supplementary Fig. S9, Table S11). F_ST_ estimated between pairs of the three, four and five groups computed from all datasets showed either great (0.150–0.250) or very great (> 0.250) genetic differentiation^65^, which varied from 0.154 to 0.819 between pairs of the 5 groups, from 0.261 to 0.827 between pairs of the four groups and from 0.453 to 0.785 between pairs of the three groups (Supplementary Table S12). The extent of molecular variance partitioned within and among groups as well as the extent of genetic differentiation between pairs of groups was consistently similar irrespective of the sampling methods and datasets

## Discussion

### Genetic diversity within and between accessions

In most genomics studies of self-pollinating species held in genebank collections, each accession is often represented by genotype data taken from a single randomly sampled individual^19,25,66^; this is usually done before or after one or more generations of seed purification using single seed descent under field or screen-house conditions^67^. Accessions conserved at a given genebank may have been originally collected as populations, which are often heterogeneous with a larger plant to plant variation. Most genebanks minimize such high levels of intra-accession variation by purifying seed lots to make them acceptable for genetic and pre-breeding studies^68^. In a recent example, our group at the AfricaRice genotyped 3,245 accessions belonging to *O. barthii* (115), *O. sativa* (772) and *O. glaberrima* (2,358) with 26,073 physically mapped SNPs^21^, with each accession represented by a single plant after seed purification^25^. There are, however, concerns regarding the development of purified seed lots and/or use of a single individual to represent a genebank collection, especially in landraces and wild accessions. First, seed purification of thousands of accessions conserved at a given genebank incurs additional financial resources, personnel, time, and space. Since each accession can be then split into two or more new seed lots after purification, these additional resources are needed not only for purification but also for managing/maintaining the purified seed lots^68^. Second, most genebanks do not have clear strategies to manage the purified germplasm sets (seed lots). Third, the genotypic data generated from a single individual per accession with or without seed purification may not capture the genetic variation available within a given collection. Finally, genotyping of bulks of multiple individuals per accession for genomic studies in selfing species has been suggested^17^ but is not yet commonly used in inbreeding species, although is it commonly used for similar purposes in cross-pollinating species^37,38,61,69,70^. To the best of our knowledge, therefore, this is the first extensive and systematic study to generate well-designed empirical data for assessing the level and distribution of intra- and inter-accession genetic diversity across different leaf/DNA sampling methods in three rice species and different genetic backgrounds using genome-wide SNPs.

Overall, the various types of univariate and multivariate analyses performed in the present study revealed relatively consistent patterns of marker polymorphisms, heterozygosity, intra-accession, and inter-accession diversity indices, genetic dissimilarity, population structure, and genetic differentiation irrespective of the sampling methods. Of the 1,527 DNA samples used in the present study, (1) 96.5% of the single plant DNA samples originating from the same accession clustered together as expected and only 3.5% of the individuals clustered with other accessions; (2) the two bulks of 5 and 10 plants within an accession consistently clustered with the 15 single plant samples in 95.6% of the 90 accessions; (3) θ and π computed as measures of genetic diversity within each accession were smaller than 0.06 in 81 of the 90 accessions, which suggests greater than expected intra-accession diversity within 10% of the accessions; (4) there were highly comparable patterns of polymorphisms (98.8–99.9%) among all datasets irrespective of the sampling methods (Supplementary Table S2); and (5) there were high to very high correlations among distance matrices computed from the different datasets generated for all 90 accessions, except for *O. glaberrima* (see below) when analyses were done on a priori known groups.

Although our genotypic data for the 5–15 individuals per accession did not provide strong justification for compensating the 4–14-fold increase in sample preparation and genotyping costs compared to using a single plant, we observed some level of disagreement between datasets within some accessions, which included *O. glaberrima* (1 accession), *O. barthii* (2 accessions), and *O. sativa* adapted to the lowland (6 accessions) and upland (7 accessions) ecologies. Pairwise differences among the multiple individuals of these accessions have been summarized in Figs. 3, 4 and 5 and Supplementary Figs. S1-S2, which demonstrated a relatively larger intra-accession genetic diversity due to a few individuals that are equivalent to inter-accession diversity; this has also been seen in other studies of self-pollinated genebank material^5,19^. In a genomic study of barley genebank accessions using GBS, there were 32 accessions represented by 10 individuals each that revealed varying degrees of intra-accession diversity. About 34% of the 32 barley accessions showed very little intra-accession diversity, while 16% showed an intra-accession divergence that was equivalent to inter-accession diversity^19^. Using morphological and isozyme markers, intra-accessions genetic diversity has also been reported in another study of barley landraces conserved in genebanks for 10–72 years^5^. In most accessions, our results obtained from the two datasets corresponding to the 5 and 10 single plants were highly similar to those of the 15 individuals.

To capture the larger intra-accession diversity observed within some of the accessions, we recommend a single bulk of either 5 or 10 individuals instead of genotyping 5–15 plants individually per accession; this is evident from the very high positive correlations (0.871 ≤ r ≤ 0.995) between distance matrices computed from Set-2, Set-3, Set-4, Set-11 and Set-12 (Supplementary Table S9). The genotyping cost of a bulk (of 5 or 10 plants) would be the same as a single individual and 4–14-fold cheaper than genotyping 5–15 plants individually (see below for details), but bulks should help capture more intra-accession diversity than a single plant. We recommend, however, that the bulks be made by pooling approximately equal leaf tissue from every individual and only use up to 15 plants per bulk in order not to dilute rare alleles when more plants are bulked per accession^38,45^. *O. glaberrima* was the only exception that showed lower correlations between distance matrices computed from the 5–15 individuals in Set-2, Set-11 and Set-12 vs. the bulks of 5 and 10 plants in Set-3 and Set-4 (0.477 ≤ r ≤ 0.690), which may either be due to the genetic background of this species and/or an ascertainment bias in the SNPs. Although ascertainment bias is minimal with genotyping by sequencing technologies, it may arise when marker data is not obtained from a random sample of the polymorphisms^71^, which could occur in the current study due to the use of the *O. sativa* spp. japonica (cv. Nipponbare) reference genome for aligning marker sequences. Some level of ascertainment bias may have also been introduced by the Random Forest^46^ imputation method used in this study, as has been reported in another study in wheat^72^.

### Genetic relationship and population structure

Overall, the different DNA sampling methods revealed very consistent patterns of genetic relationships, population structures, and genetic differentiation irrespective of species, genetic background, and predicted group memberships (Fig. 6, Supplementary Fig. S7, Fig. S8, Table S11, Table S12). However, there were some exceptions, including 3.5% of the individual samples that clustered together with other accessions of either the same or a different species and two *O. barthii* accessions that showed an inconsistent pattern of clustering across datasets in the phylogenetic trees (Fig. 6, Supplementary Fig. S7). The mis-clustered samples are likely outliers due to errors during seed handling, sample preparation and/or genotyping^17,26,37^. Mislabeling, misclassification (misidentification), and mixing of samples are common problems in genebanks^15^ and have been reported in several species, including multiple *Oryza* species^26,73,74^, *Dioscorea* spp.^75^, *Brassica* spp ^76^. and *Solanum* spp.^77^. The percentage of mislabeled or misclassified samples reported in the literature is highly variable depending on sample size, the species, and the methods used for assessing the error rates, which varied from 3 to 21%^26,73–77^. In one of our recent studies, we found that 3.1% of 3,134 of accessions from four rice species were either mislabeled or misclassified^26^, which can easily be checked using a subset of the diagnostic SNPs that we developed in the previous study. Misclassification and mislabeling not only restrict the effective use of the germplasm for various purposes but also provide an erroneous estimate of intra-accession and inter-accession genetic diversity; in such cases, the presence of larger intra-accession genetic diversity can be an indication of errors rather than genetics/biological.

Both Robinson-Foulds and SPR distances computed as measures of disagreements between a pair of phylogenies revealed that the phylogenetic tree constructed from the 15 individuals in Set-2 had the highest concordance with those in Set-12 (RF = 0.15, SPR = 7), followed by Set-11 (RF = 0.37, SPR = 21). All other values suggest a low to moderate concordance among pairs of phylogenies (Supplementary Table S10). It should be noted, however, that the concordance among pairs of phylogenies may be confounded by multiple factors, including topological features (the number of shared/non-shared subtrees) between a pair of trees, path length information (finding the nearest neighbor interchange to transform one tree into another), edge weights, and branch scores^58,78–80^, all of which are of little relevance in characterizing genebank collections. In germplasm characterization, phylogenetic trees are primarily used to understand the broader pattern of evolutionary relationships; the level of genetic divergence; the definition of groups (populations or sub-populations); the selection of subsets of accessions that capture the genetic variation of a given group; and the identification of potential duplicates. In such cases, it is often difficult for genetic resource scientists to determine the true historical relationships between any groups of accessions other than using the bootstrapping method for assessing the accuracy or confidence in phylogenetic trees^81^. Although some studies advocate for bootstrapping, other studies believe that bootstrap values are a poor measure of repeatability^82^ depending on (1) the methods used for computing similarity/dissimilarity matrices; (2) the algorithm/methodology implemented in constructing the phylogenetic trees and for assessing disagreements between pairs of trees^80^, and (3) the lack of clear-cut threshold bootstrap values (which vary from 70 to 100%) that is used to judge whether a given node is good or not. Furthermore, displaying nodal support bootstrap values is difficult for large datasets^83,84^, which are typical of large-scale germplasm curation and characterization studies.

### Cost and technical feasibility

The availability of high throughput and relatively low-cost NGS technologies have provided genebank researchers a better opportunity to explore the genetic potential of their collections^15^. The current DNA extraction and genotyping cost of a single sample with DArTseq technology through a commercial vendor range from the US $22 to $30 per sample (the actual cost depends on sample size), which returns between 22,000 to 47,000 polymorphic SNPs in rice. A small sample size can underestimate genetic diversity parameters, and excessive sampling inflates costs^2^. Sampling 5–15 plants per accession instead of one provides more intra-accession information, but it does inflate sampling, DNA extraction, and genotyping cost 4–14-fold. In the present study, for example, genotyping of 5, 10, and 15 individuals incurred an additional cost per accession of US $88, $198, and $308, respectively. Currently, the AfricaRice genebank holds 21,300 accessions (https://www.genesys-pgr.org/), which would cost ~ US $2.4 and $7.1 million for genotyping 5 and 15 individuals per accession, respectively, compared to $468,600 for genotyping either a single individual or a bulk. Because we arrived at broadly similar conclusions regardless of sampling methods for most applications, we do not believe the additional information obtained by genotyping 5–15 individuals justify the multi-fold increase in cost. Furthermore, sampling of 5–15 individuals per accession across thousands of accessions raises another concern in that the technical feasibility of sampling, processing, and tracking so many individuals, followed by managing the high-density genotypic data, will be extremely challenging^85–87^. Recently, our team genotyped 4,115 rice accessions with ~ 32,000 SNPs using DArTseq, which generated 650-megabytes of data. If each accession had been represented by 5–15 individuals, the total number of samples would have been 21–62 thousands and approximately 3.2–9.8 gigabytes of data, and data analysis with existing statistical programs would have been extremely challenging. Genotyping of all 21,300 accessions with 5–15 individuals could lead to a daunting file size of 16.8 to 50.5-gigabytes.

## Conclusions

Using high-density DArTseq genotype data generated with the Illumina NGS technology, we assessed six leaf (DNA) sampling methods to determine if an obvious advantage in genotyping multiple individuals per accessions existed to justify the multi-fold increase in cost and technical complexity of handling/managing large number of samples per accession as compared to genotyping either a randomly selected individual or a bulk. Overall, we arrived at broadly similar conclusions in terms of overall SNPs polymorphism and heterozygosity/heterogeneity; molecular diversity indices within and between accessions and groups; the genetic dissimilarity between accessions and groups; population structure; and genetic differentiation. Genotyping 5–15 individuals per accession provided better information for understanding not only the level of intra-accession genetic diversity but also for detecting outliers over genotyping a randomly selected individual; however, the additional information obtained was not enough to justify the 4–14-fold increase in cost and technical challenges in managing the large-sample size associated with genebank genomics studies. Both Robinson-Foulds and SPR distances computed as measures of disagreements between a pair of phylogenies revealed that the phylogenetic tree constructed from the 15 individuals in Set-2 had the highest concordance with those in Set-12 (10 individuals), followed by Set-11 (5 individuals), suggesting that at least 5–10 plants should be genotyped per accession individually or in a bulk. Furthermore, the identification of suspected outliers in 26 of the 90 accessions, which accounted for 3.5–10.0% of the single DNA samples in Set-2, lead us to recommend genotyping of 5–10 plants individually or in a bulk instead of a single individual per accession. Results from this study provide highly useful information to other researchers involved in genetic resources characterization using genebank genomics.

## Supplementary information

Supplementary Figures

Supplementary Table S1

Supplementary Table S2

Supplementary Table S3

Supplementary Table S4

Supplementary Table S5

Supplementary Table S6

Supplementary Table S7

Supplementary Table S8

Supplementary Table S9

Supplementary Table S10

Supplementary Table S11

Supplementary Table S12

Supplementary information title

## Data Availability

All relevant data are within the paper and its Supporting Information Files.

## References

[1] Khanlou KM, Vandepitte K, Asl LK, Van Bockstaele E (2011). Towards an optimal sampling strategy for assessing genetic variation within and among white clover (*Trifolium repens* L.) cultivars using AFLP. Genet. Mol. Biol..

[2] Suzuki J-I, Herben T, Maki M (2004). An under-appreciated difficulty: sampling of plant populations for analysis using molecular markers. Evol. Ecol..

[3] van Treuren R, van Hintum TJL (2001). Identification of intra-accession genetic diversity in selfing crops using AFLP markers: implications for collection management. Genet. Resour. Crop Evol..

[4] van Hintum TJL, van de Wiel CCM, Visser DL, van Treuren R, Vosman B (2007). The distribution of genetic diversity in a Brassica oleracea gene bank collection related to the effects on diversity of regeneration, as measured with AFLPs. Theor. Appl. Genet..

[5] Parzies HK, Spoor W, Ennos RA (2000). Genetic diversity of barley landrace accessions (*Hordeum vulgare* spp. vulgare) conserved for different lengths of time in ex situ gene banks. Heredity.

[6] Bryan GJ, McLean K, Waugh R, Spooner DM (2017). Levels of intra-specific AFLP diversity in tuber-bearing potato species with different breeding systems and ploidy levels. Front. Genet..

[7] Lowe AJ, Thorpe W, Teale A, Hanson J (2003). Characterisation of germplasm accessions of Napier grass (*Pennisetum purpureum* and *P. purpureum* × *P. glaucum* hybrids) and comparison with farm clones using RAPD. Genet. Resour. Crop Evol..

[8] Sudupak MA (2004). Inter and intra-species inter simple sequence repeat (ISSR) variations in the genus Cicer. Euphytica.

[9] Alansi S, Tarroum M, Al-Qurainy F, Khan S, Nadeem M (2016). Use of ISSR markers to assess the genetic diversity in wild medicinal *Ziziphus spina-christi* (L.) Willd. collected from different regions of Saudi Arabia. Biotechnol. Biotechnol. Equip..

[10] El-Esawi MA, Germaine K, Bourke P, Malone R (2016). Genetic diversity and population structure of Brassica oleracea germplasm in Ireland using SSR markers. C. R. Biol..

[11] Semagn K, Bjornstad A, Ndjiondjop MN (2006). An overview of molecular marker methods for plants. Afr. J. Biotechnol..

[12] Idury RM, Cardon LR (1997). A simple method for automated allele binning in microsatellite markers. Genome Res..

[13] Ginot F, Bordelais I, Nguyen S, Gyapay G (1996). Correction of some genotyping errors in automated fluorescent microsatellite analysis by enzymatic removal of one base overhangs. Nucleic Acids Res..

[14] Ghosh S (1997). Methods for precise sizing, automated binning of alleles, and reduction of error rates in large-scale genotyping using fluorescently labeled dinucleotide markers. Genome Res..

[15] McCouch SR, McNally KL, Wang W, Hamilton RS (2012). Genomics of gene banks: a case study in rice. Am. J. Bot..

[16] Mascher M (2019). Genebank genomics bridges the gap between the conservation of crop diversity and plant breeding. Nat. Genet..

[17] Singh N (2019). Efficient curation of genebanks using next generation sequencing reveals substantial duplication of germplasm accessions. Sci. Rep..

[18] Hu Z, Olatoye MO, Marla S, Morris GP (2019). An integrated genotyping-by-sequencing polymorphism map for over 10,000 sorghum genotypes. Plant Genome.

[19] Milner SG (2019). Genebank genomics highlights the diversity of a global barley collection. Nat. Genet..

[20] Wegary D (2019). Molecular diversity and selective sweeps in maize inbred lines adapted to African highlands. Sci. Rep..

[21] Ndjiondjop MN (2019). Comparisons of molecular diversity indices, selective sweeps and population structure of African rice with its wild progenitor and Asian rice. Theor. Appl. Genet..

[22] Lv S (2018). Genetic control of seed shattering during African rice domestication. Nat. Plants.

[23] Gouesnard B (2017). Genotyping-by-sequencing highlights original diversity patterns within a European collection of 1191 maize flint lines, as compared to the maize USDA genebank. Theor. Appl. Genet..

[24] Muktar MS (2019). Genotyping by sequencing provides new insights into the diversity of Napier grass (*Cenchrus purpureus*) and reveals variation in genome-wide LD patterns between collections. Sci. Rep..

[25] Ndjiondjop M-N (2017). Genetic variation and population structure of *Oryza glaberrima* and development of a mini-core collection using DArTseq. Front. Plant Sci..

[26] Ndjiondjop MN (2018). Development of species diagnostic SNP markers for quality control genotyping in four rice (*Oryza* L) species. Mol. Breed..

[27] Ertiro BT (2015). Comparison of kompetitive allele specific PCR (KASP) and genotyping by sequencing (GBS) for quality control analysis in maize. BMC Genom..

[28] Sansaloni C (2011). Diversity arrays technology (DArT) and next-generation sequencing combined: genome-wide, high throughput, highly informative genotyping for molecular breeding of Eucalyptus. BMC Proc..

[29] Ndjiondjop MN (2018). Assessment of genetic variation and population structure of diverse rice genotypes adapted to lowland and upland ecologies in Africa using SNPs. Front. Plant Sci..

[30] Semon M, Nielsen R, Jones MP, McCouch SR (2005). The population structure of African cultivated rice *Oryza glaberrima* (Steud.): evidence for elevated levels of linkage disequilibrium caused by admixture with *O. sativa* and ecological adaptation. Genetics.

[31] Cubry P (2018). The rise and fall of African rice cultivation revealed by analysis of 246 new genomes. Curr. Biol..

[32] Barnaud A, Trigueros G, McKey D, Joly HI (2008). High outcrossing rates in fields with mixed sorghum landraces: How are landraces maintained?. Heredity.

[33] Phan PDT, Kageyama H, Ishikawa R, Ishii T (2012). Estimation of the outcrossing rate for annual Asian wild rice under field conditions. Breed. sci..

[34] Michelmore RW, Paran I, Kesseli RV (1991). Identification of markers linked to disease-resistance genes by bulked segregant analysis: a rapid method to detect markers in specific genomic regions by using segregating populations. Proc. Natl. Acad. Sci. U.S.A..

[35] Giovannoni JJ, Wing RA, Ganal MW, Tanksley SD (1991). Isolation of molecular markers from specific chromosomal intervals using DNA pools from existing mapping populations. Nucleic Acids Res..

[36] Semagn K, Bjornstad A, Xu Y (2010). The genetic dissection of quantitative traits in crops. Electron. J. Biotechnol..

[37] Warburton ML (2010). Toward a cost-effective fingerprinting methodology to distinguish maize open-pollinated varieties. Crop Sci..

[38] Dubreuil P, Warburton M, Chastanet M, Hoisington D, Charcosset A (2006). More on the introduction of temperate maize into Europe: large-scale bulk SSR genotyping and new historical elements. Maydica.

[39] Wu Y (2016). Molecular characterization of CIMMYT maize inbred lines with genotyping-by-sequencing SNPs. Theor. Appl. Genet..

[40] Song J, Li Z, Liu Z, Guo Y, Qiu LJ (2017). Next-generation sequencing from bulked-segregant analysis accelerates the simultaneous identification of two qualitative genes in soybean. Front. Plant Sci..

[41] Wambugu P, Ndjiondjop MN, Furtado A, Henry R (2018). Sequencing of bulks of segregants allows dissection of genetic control of amylose content in rice. Plant Biotechnol. J..

[42] Dong W, Wu D, Li G, Wu D, Wang Z (2018). Next-generation sequencing from bulked segregant analysis identifies a dwarfism gene in watermelon. Sci. Rep..

[43] Gyawali A, Shrestha V, Guill KE, Flint-Garcia S, Beissinger TM (2019). Single-plant GWAS coupled with bulk segregant analysis allows rapid identification and corroboration of plant-height candidate SNPs. BMC Plant Biol..

[44] Vikram P, Swamy BPM, Dixit S, Ahmed HA (2012). Bulk segregant analysis: an effective approach for mapping consistent-effect drought grain yield QTLs in rice. Field Crops Res..

[45] Reyes-Valdés MH (2013). Analysis and optimization of bulk DNA sampling with binary scoring for germplasm characterization. PLoS ONE.

[46] Breiman L (2001). Random forests. Mach. Learn..

[47] Liaw A, Wiener M (2002). Classification and regression by randomforest. R News.

[48] Mantel N (1967). The detection of disease clustering and a generalized regression approach. Cancer Res..

[49] Rholf FJ (1993). NTSYS-pc, Numerical Taxonomy and Multivariate Analysis System.

[50] Baloch FS (2017). A whole genome DArTseq and SNP analysis for genetic diversity assessment in durum wheat from central fertile crescent. PLoS ONE.

[51] Melville J (2017). Identifying hybridization and admixture using SNPs: application of the DArTseq platform in phylogeographic research on vertebrates. R. Soc. Open Sci..

[52] Bradbury PJ (2007). TASSEL: software for association mapping of complex traits in diverse samples. Bioinformatics.

[53] Kumar S, Stecher G, Li M, Knyaz C, Tamura K (2018). MEGA X: molecular evolutionary genetics analysis across computing platforms. Mol. Biol. Evol..

[54] Pritchard JK, Stephens M, Donnelly P (2000). Inference of population structure using multilocus genotype data. Genetics.

[55] Excoffier L, Lischer HEL (2010). Arlequin suite ver 3.5: a new series of programs to perform population genetics analyses under Linux and Windows. Mol. Ecol. Resour..

[56] Lischer HEL, Excoffier L (2012). PGDSpider: an automated data conversion tool for connecting population genetics and genomics programs. Bioinformatics.

[57] Robinson O, Dylus D, Dessimoz C (2016). Phylo.io: Interactive viewing and comparison of large phylogenetic trees on the web. Mol. Biol. Evol..

[58] Robinson DF, Foulds LR (1981). Comparison of phylogenetic trees. Math. Biosci..

[59] De Oliveira Martins L, Mallo D, Posada D (2016). A Bayesian supertree model for genome-wide species tree reconstruction. Syst. Biol..

[60] de Oliveira Martins L, Leal ÉK, Hirohisa (2008). Phylogenetic detection of recombination with a Bayesian prior on the distance between trees. PLoS ONE.

[61] Semagn K (2012). Molecular characterization of diverse CIMMYT maize inbred lines from eastern and southern Africa using single nucleotide polymorphic markers. BMC Genom..

[62] Excoffier L, Smouse PE, Quattro JM (1992). Analysis of molecular variance inferred from metric distances among DNA haplotypes: application to human mitochondrial DNA restriction data. Genetics.

[63] Holsinger KE, Weir BS (2009). Genetics in geographically structured populations: defining, estimating and interpreting FST. Nat. Rev. Genet..

[64] Bah S, van der Merwe R, Labuschagne MT (2017). Estimation of outcrossing rates in intraspecific (*Oryza sativa*) and interspecific (*Oryza sativa* × *Oryza glaberrima*) rice under field conditions using agro-morphological markers. Euphytica.

[65] Wright S (1978). Evolution and the Genetics of Populations: Variability within and Among Natural Populations vol. 4.

[66] Singh S (2018). Harnessing genetic potential of wheat germplasm banks through impact-oriented-prebreeding for future food and nutritional security. Sci. Rep..

[67] Project, T. R. G (2014). The 3,000 rice genomes project. GigaScience.

[68] Anglin NL, Amri A, Kehel Z, Ellis D (2018). A case of need: Linking traits to genebank accessions. Biopreserv. Biobank..

[69] Lu Y (2009). Molecular characterization of global maize breeding germplasm based on genome-wide single nucleotide polymorphisms. Theor. Appl. Genet..

[70] Warburton ML (2005). Genetic characterization of 218 elite CIMMYT maize inbred lines using RFLP markers. Euphytica.

[71] Heslot N, Rutkoski J, Poland J, Jannink J-L, Sorrells ME (2013). Impact of marker ascertainment bias on genomic selection accuracy and estimates of genetic diversity. PLoS ONE.

[72] Brandariz SP (2016). Ascertainment bias from imputation methods evaluation in wheat. BMC Genom..

[73] Orjuela J (2014). An extensive analysis of the African rice genetic diversity through a global genotyping. Theor. Appl. Genet..

[74] Buso GSC, Rangel PHN, Ferreira ME (2001). Analysis of random and specific sequences of nuclear and cytoplasmic DNA in diploid and tetraploid American wild rice species (*Oryza* spp.). Genome.

[75] Girma G, Korie S, Dumet D, Franco J (2012). Improvement of accession distinctiveness as an added value to the global worth of the yam (*Dioscorea* spp.) genebank. Int. J. Conserv. Sci..

[76] Mason AS (2015). High-throughput genotyping for species identification and diversity assessment in germplasm collections. Mol. Ecol. Resour..

[77] Ellis D (2018). Genetic identity in genebanks: application of the SolCAP 12K SNP array in fingerprinting and diversity analysis in the global in trust potato collection. Genome.

[78] Choi K, Gomez SM (2009). Comparison of phylogenetic trees through alignment of embedded evolutionary distances. BMC Bioinform..

[79] Hein J, Jiang T, Wang L, Zhang K (1996). On the complexity of comparing evolutionary trees. Discrete Appl. Math..

[80] Som A (2014). Causes, consequences and solutions of phylogenetic incongruence. Brief. Bioinform..

[81] Felsenstein J (1985). Confidence limits on phylogenies: an approach using the bootstrap. Evolution.

[82] Hillis DM, Bull JJ (1993). An empirical test of bootstrapping as a method for assessing confidence in phylogenetic analysis. Syst. Biol..

[83] Soltis PS, Soltis DE (2003). Applying the bootstrap in phylogeny reconstruction. Stat. Sci..

[84] Sanderson MJ, Wojciechowski MF (2000). Improved bootstrap confidence limits in large-scale phylogenies, with an example from neo-astragalus (Leguminosae). Syst. Biol..

[85] Gao S (2008). Development of a seed DNA-based genotyping system for marker-assisted selection in maize. Mol. Breed..

[86] Xu Y (2017). Enhancing genetic gain in the era of molecular breeding. J. Exp. Bot..

[87] Arbelaez JD (2019). Methodology: ssb-MASS: a single seed-based sampling strategy for marker-assisted selection in rice. Plant Methods.

