# Supplementary material for: Comparisons of sampling methods for assessing intra- and inter-accession genetic diversity in three rice species using genotyping by sequencing
Source: Sci Rep. 2020 Aug 19;10:13995. doi: 10.1038/s41598-020-70842-0 (PMC7438528; doi:10.1038/s41598-020-70842-0)
Supplement: Supplementary file 1 — Supplementary Figures [file 41598_2020_70842_MOESM1_ESM.docx]

Comparisons of DNA sampling methods for assessing intra- and inter-accession genetic diversity in three rice species using genotyping by sequencing

Arnaud C. Gouda^1^, Marie Noelle Ndjiondjop^1^*, Gustave L. Djedatin^2^, Marilyn L. Warburton^3^, Alphonse Goungoulou^1^, Sèdjro Bienvenu Kpeki^1^, Amidou N'Diaye^4^, Kassa Semagn^1*^

^1^Africa Rice Center (AfricaRice), M’bé Research Station, 01 B.P. 2551, Bouaké 01, Cote d'Ivoire.

^2^Université Nationale des Sciences, Technologies, Ingénierie et Mathématiques (UNSTIM), Abomey, Benin.

^3^Corn Host Plant Resistance Research Unit, United States Department of Agriculture-Agricultural Research Service, Mississippi State, USA.

^4^Crop Development Centre and Department of Plant Sciences, University of Saskatchewan, 51 Campus Drive, Saskatoon, SK S7N 5A8, Canada.

***Corresponding author**: [k.semagn@gmail.com](mailto:k.semagn@gmail.com), Tel +1 647 675 4064; [m.ndjiondjop@cgiar.org](mailto:m.ndjiondjop@cgiar.org), Tel +225 7645 9235
