# Supplementary material for: Comparisons of sampling methods for assessing intra- and inter-accession genetic diversity in three rice species using genotyping by sequencing
Source: Sci Rep. 2020 Aug 19;10:13995. doi: 10.1038/s41598-020-70842-0 (PMC7438528; doi:10.1038/s41598-020-70842-0)

Comparisons of sampling methods for assessing intra- and inter-accession genetic diversity in three rice species using genotyping by sequencing

Arnaud C. Gouda, Marie Noelle Ndjiondjop, Gustave L. Djedatin, Marilyn L. Warburton, Alphonse Goungoulou, Sèdjro Bienvenu Kpeki, Amidou N'Diaye, and Kassa Semagn

Supplementary Fig. S1 A plot of observed heterozygosity for 90 accessions, each represented by 15 individuals genotyped with 48,818 SNPs.

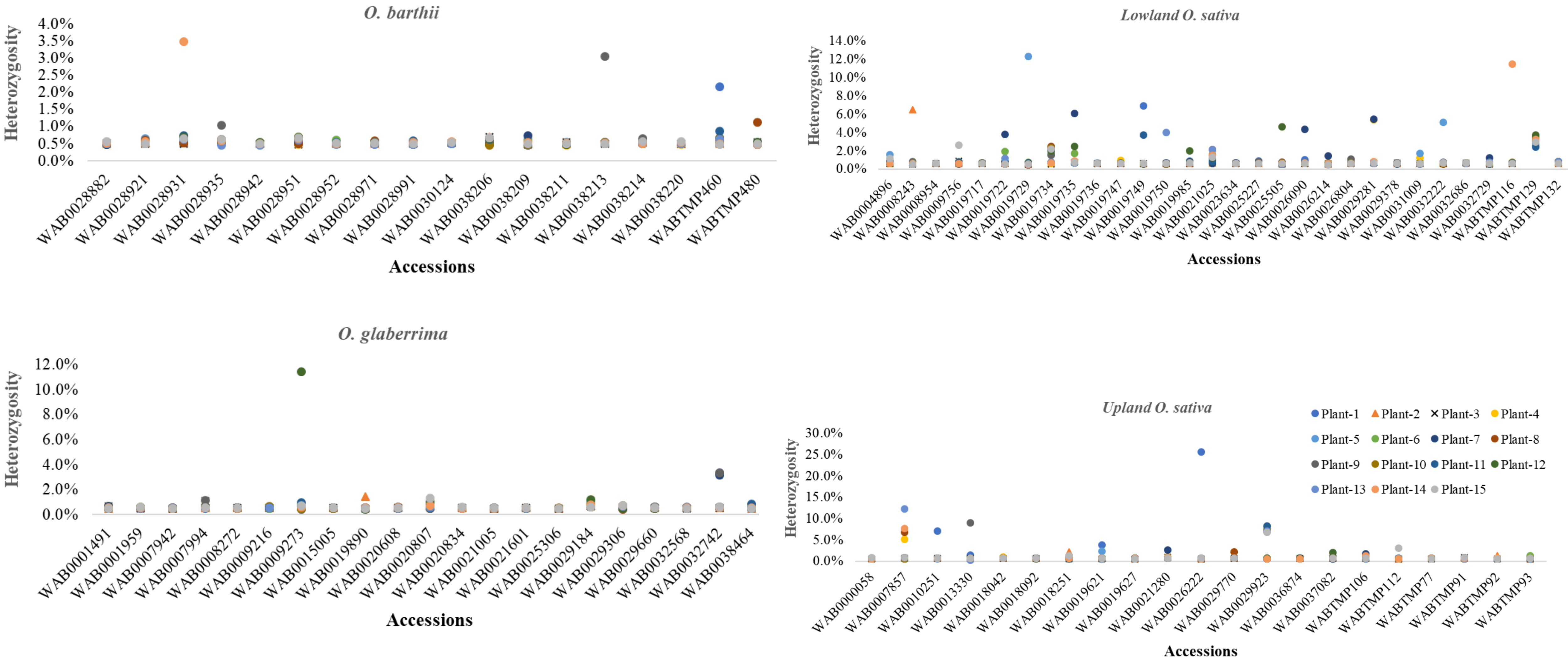

Supplementary Fig. S2 Summary of theta (θ) and nucleotide diversity (π) computed as measures of intra-accession genetic diversity across 90 accessions, each represented by 15 single plant DNA samples genotyped with 48,818 SNPs.

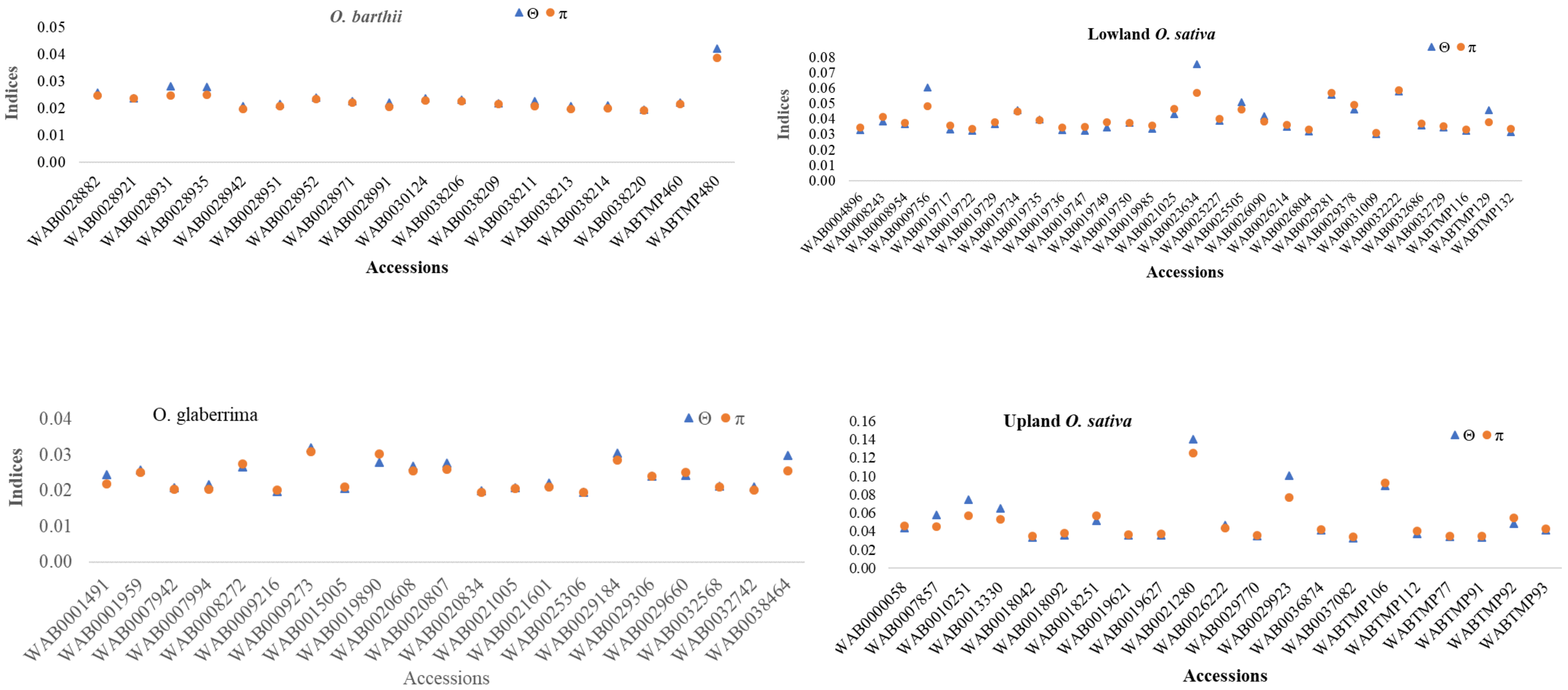

Supplementary Fig. S3 A plot of identity-by-state-based genetic distance computed in 10 accessions, each represented by 15 single plant DNA samples genotyped with 48,818 SNPs.

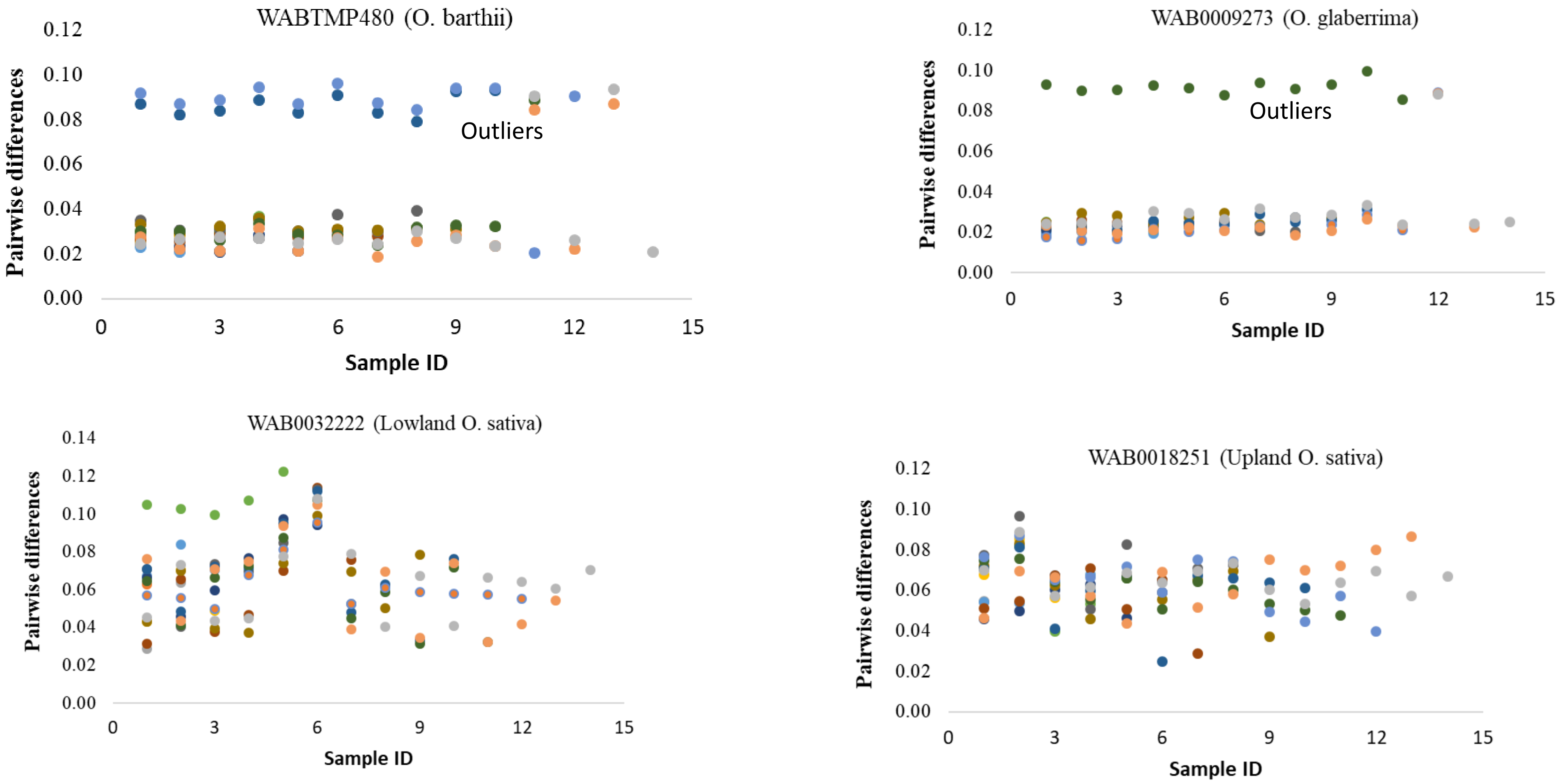

Supplementary Fig. S3 (Page 1 of 3)

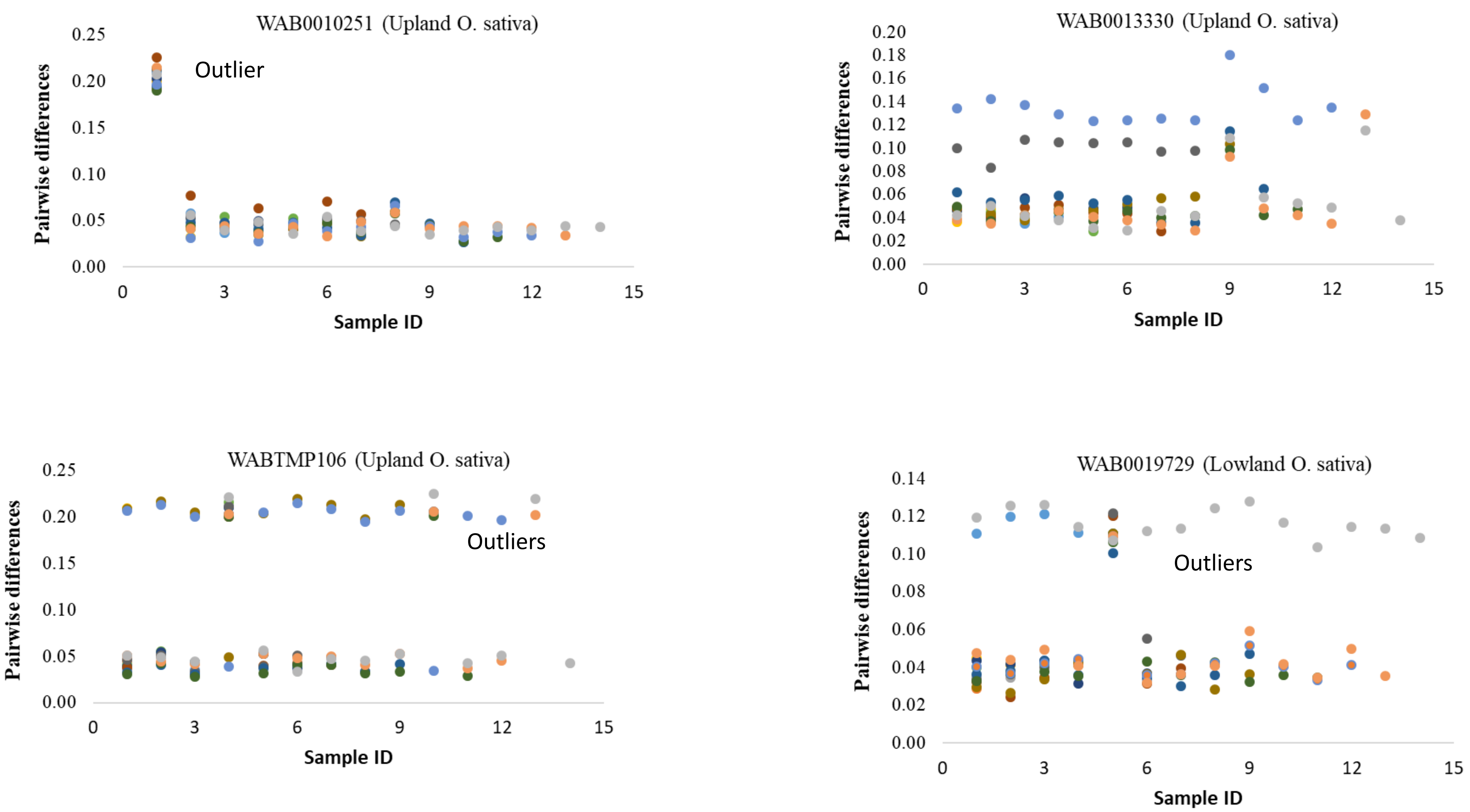

Supplementary Fig. S3 (Page 2 of 3)

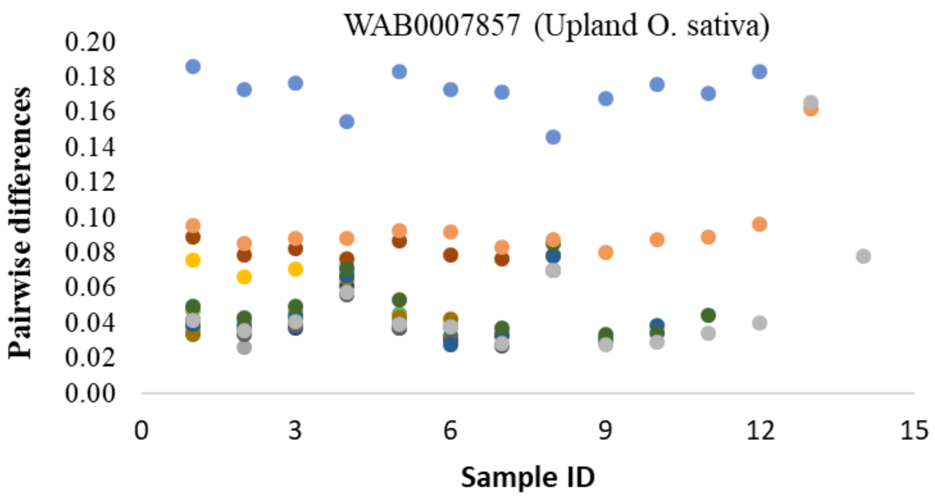

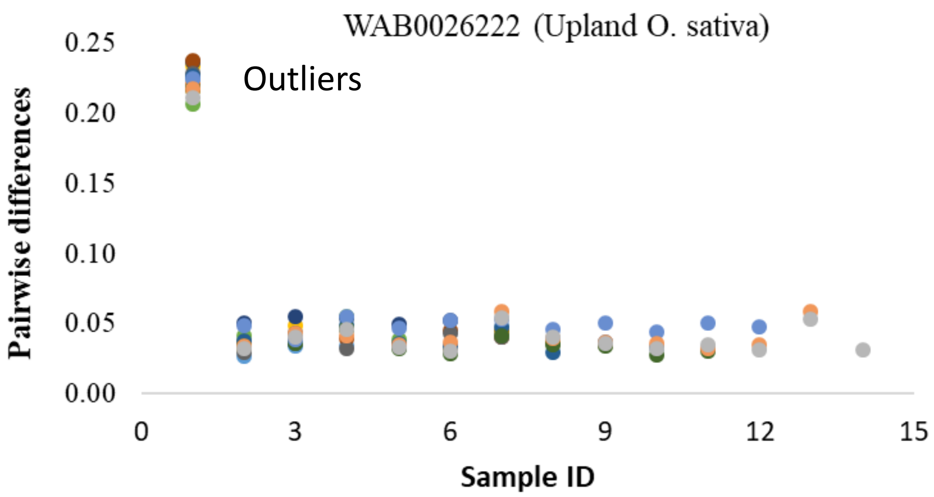

Supplementary Fig. S3 (Page 3 of 3)

Supplementary Fig. S4 Summary of observed heterozygosity across 90 accessions using eleven datasets, each consisting of 46.818 SNPs. In Set-2, Set-11, and Set-12, every accession is represented by the average heterozygosity of the 5-15 individual DNA samples. See Supplementary Table S3 for details.

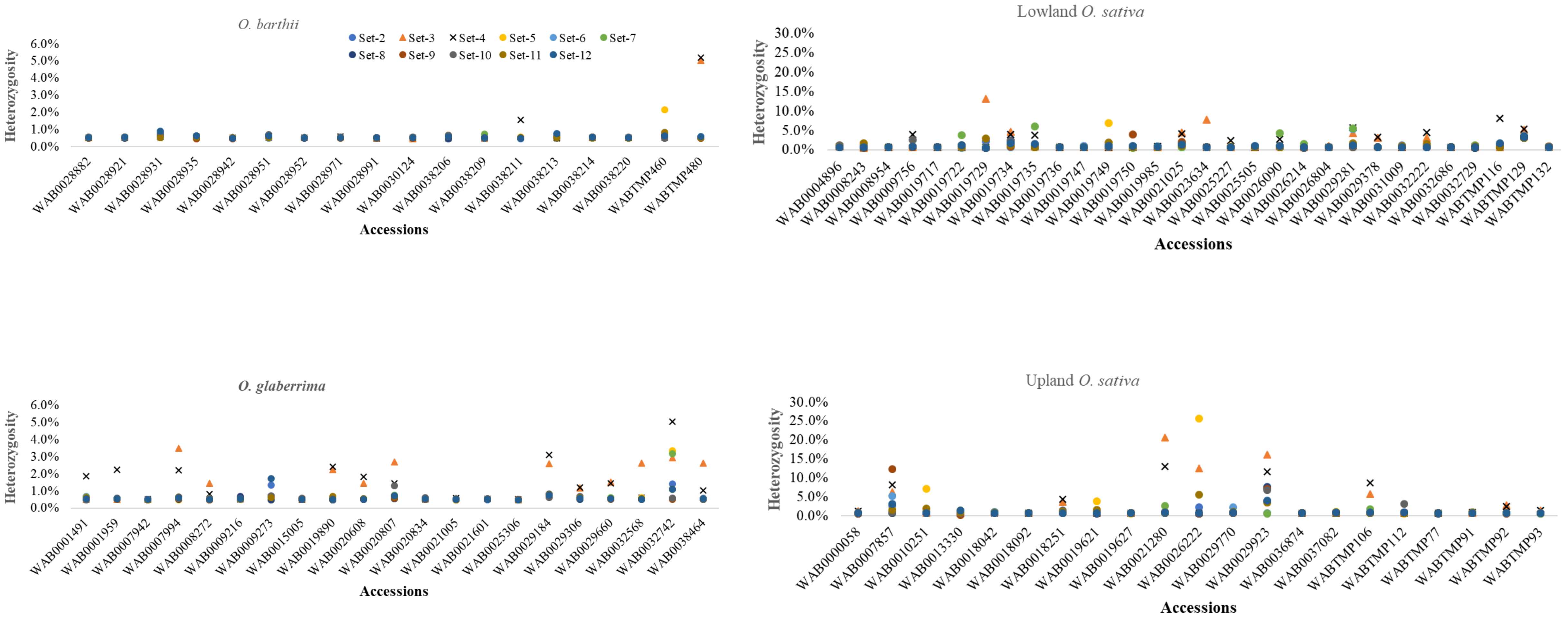

Supplementary Fig. S4 (Page 1 of 2)

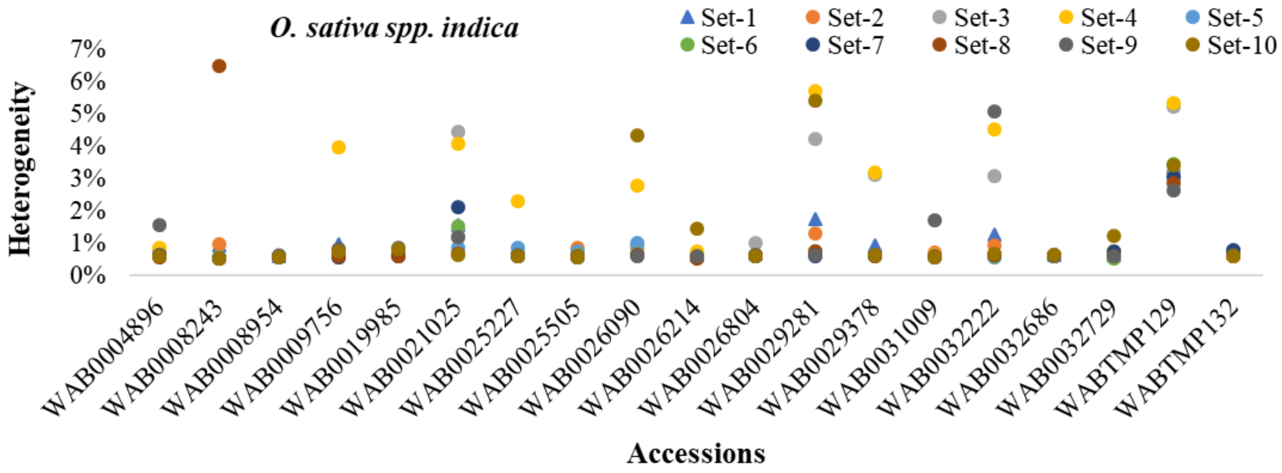

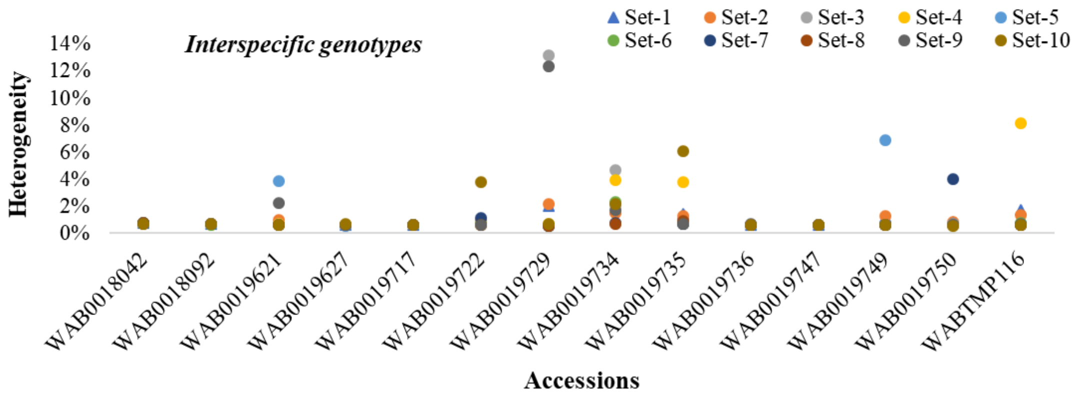

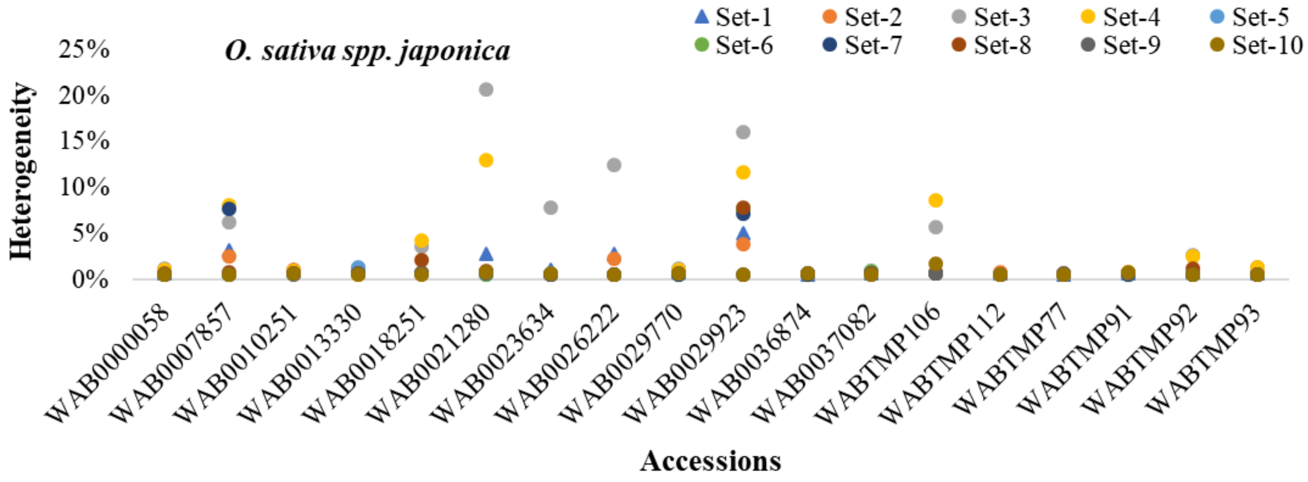

Supplementary Fig. S4 (Page 2 of 2)

Supplementary Fig. S5 Comparisons of the minimum, maximum and average maximum composite likelihood genetic distance between a pair of accessions using eleven datasets.

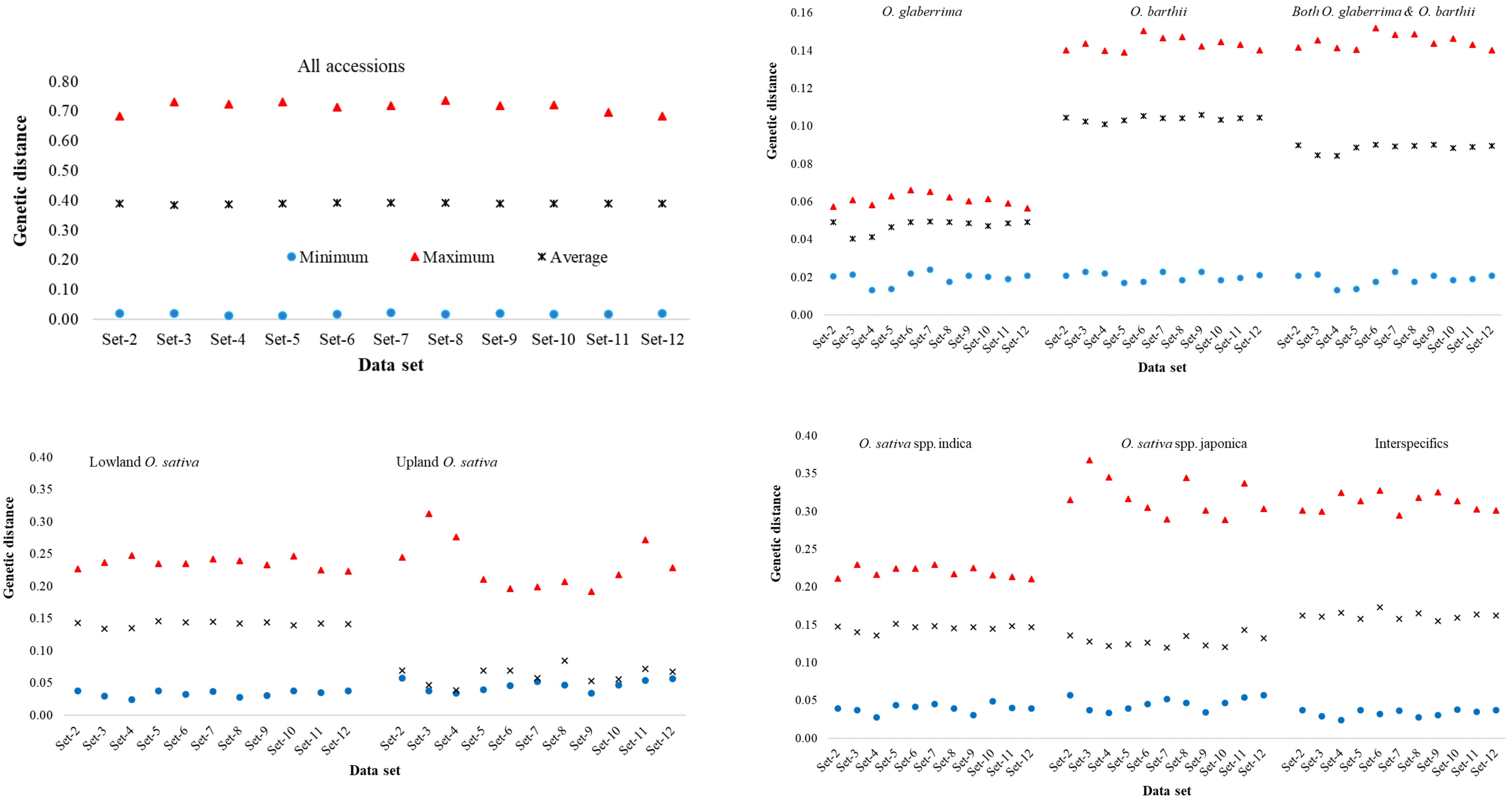

Supplementary Fig. S6 Summary of the Mantel correlations between genetic distance matrices computed from all datasets corresponding to 15 plants (Set-2), bulks of 5 plants (Set-3), bulks of 10 plants (Set-4) and randomly selected single plants out of Set-2 (Set-5 to Set-10). See Supplementary Table S9 for details.

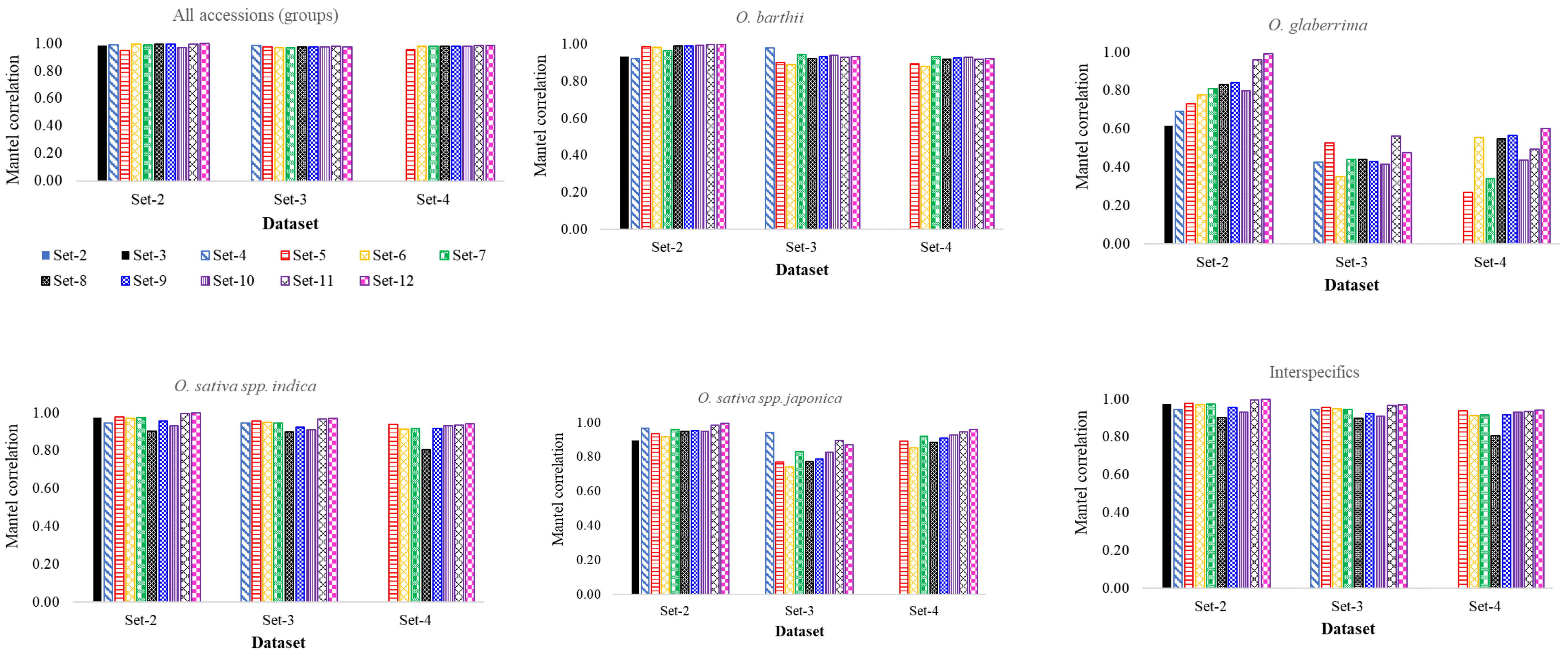

Supplementary Fig. S7 Neighbor-joining tree of 90 accessions based on maximum composite likelihood genetic distance matrices computed from 46,818 SNPs. Each accession was represented by (a) 17 DNA samples (15 individuals plus two bulks of 5 or 10 plants); (b) an average of 15 individuals (Set-2), (c) bulks of 5 plants (Set-3), (d) bulks of 10 pants (Set-4), (e-j) a randomly selected individual out of Set-2, which is repeated 6-times (Set-5 to Set-10), (k) an average of 5 individuals (Set-11) and (l) an average of 10 individuals (Set-12). Both Set-11 and Set-12 form Set-2. Accessions are colored depending on their species and/or group: *O. glaberrima* (red), *O. barthii* (blue), *O. sativa* adapted to the upland ecology (pink) and lowland ecology (black).

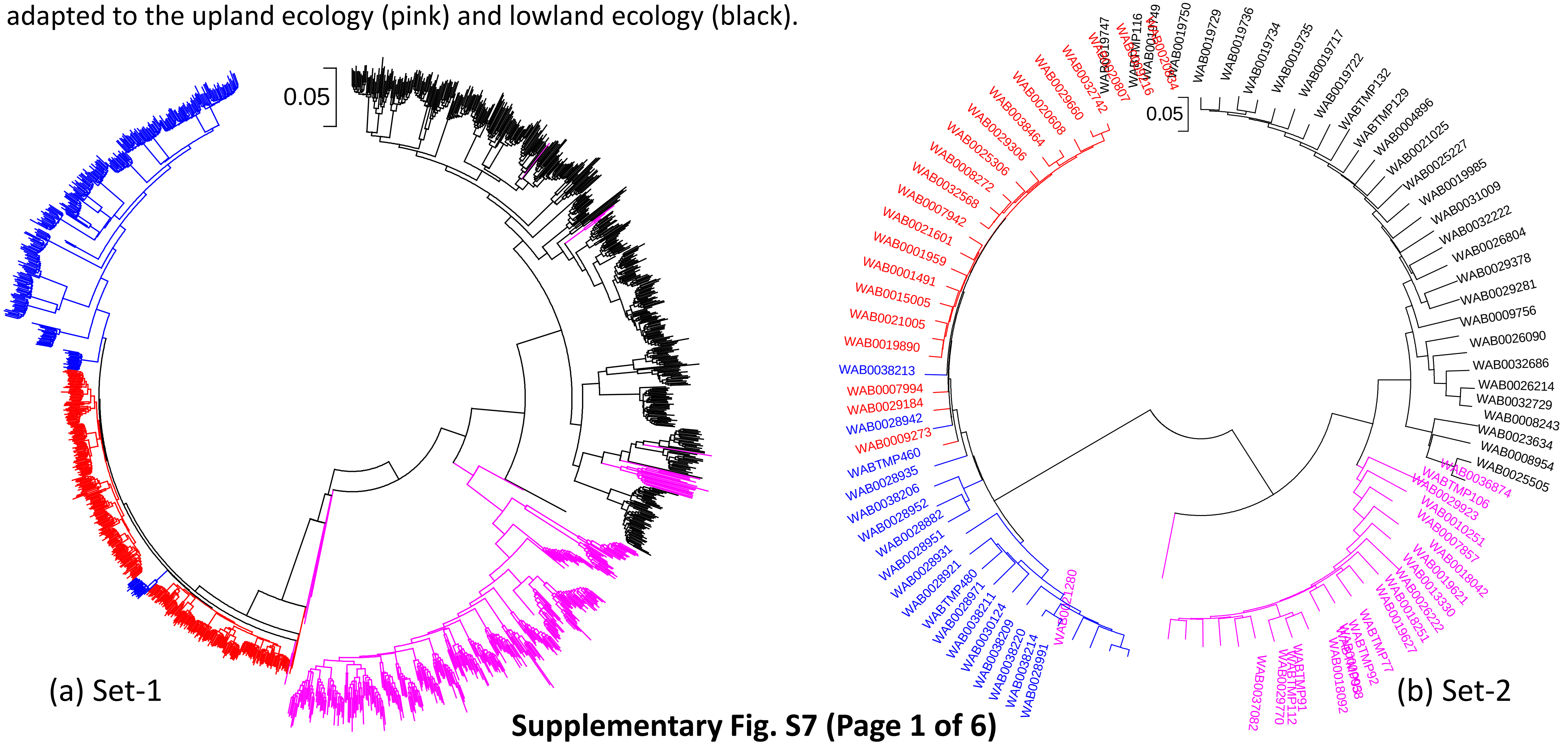

Supplementary Fig. S7 (Page 1 of 6)

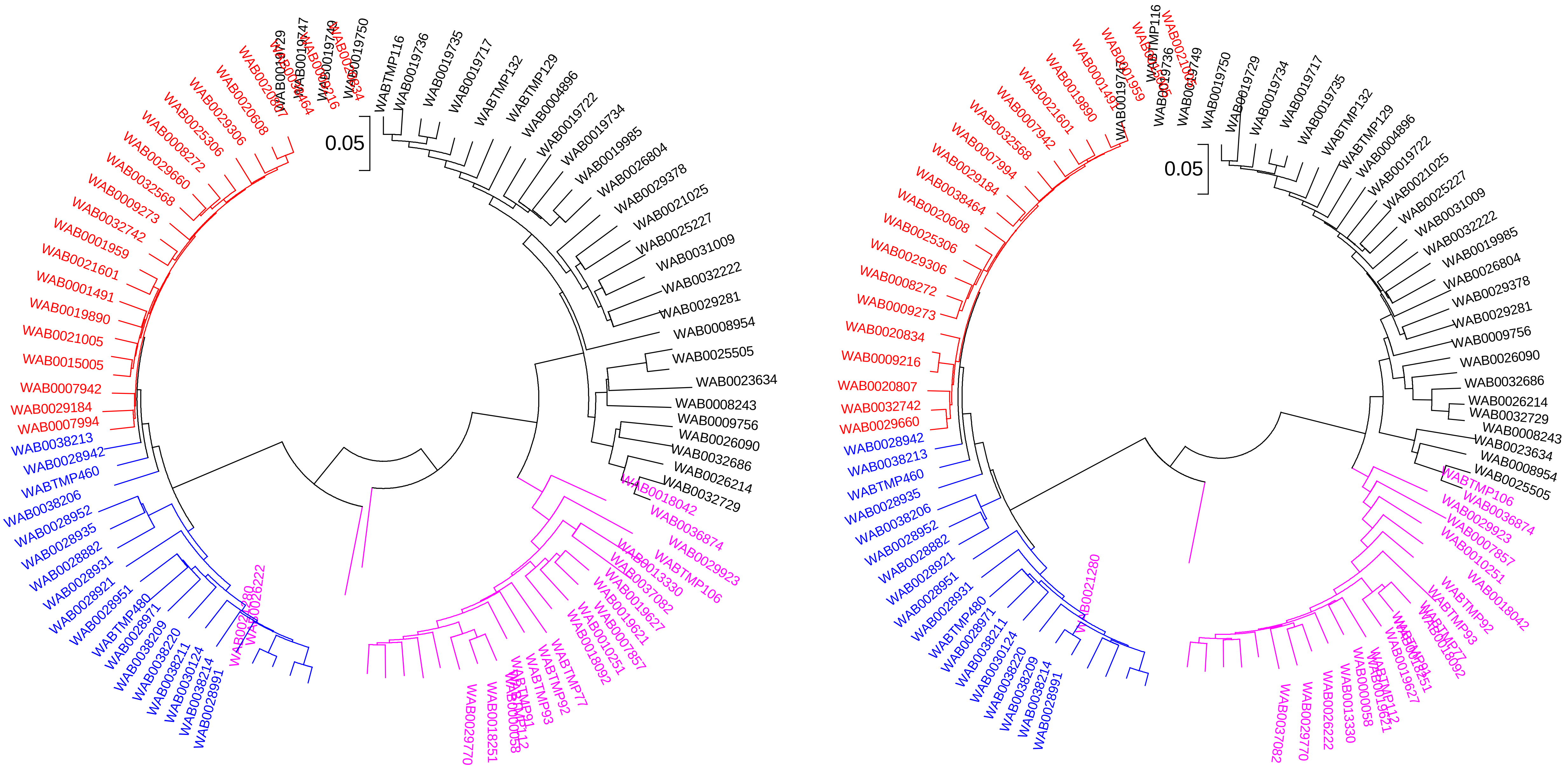

(c) Set-3 (d) Set-4

Supplementary Fig. S7 (Page 2 of 6)

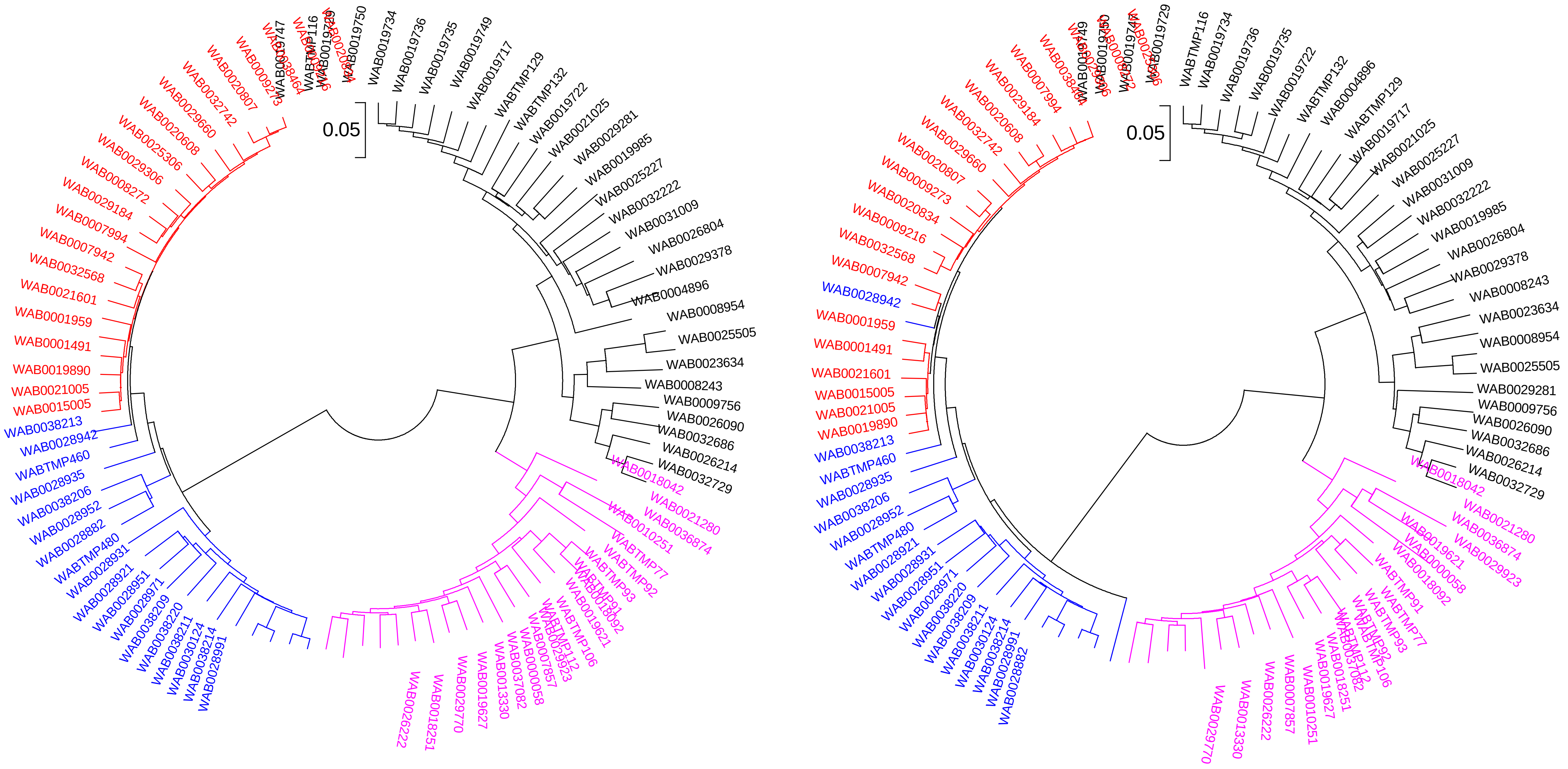

(e) Set-5

(f) Set-6

Supplementary Fig. S7 (Page 3 of 6)

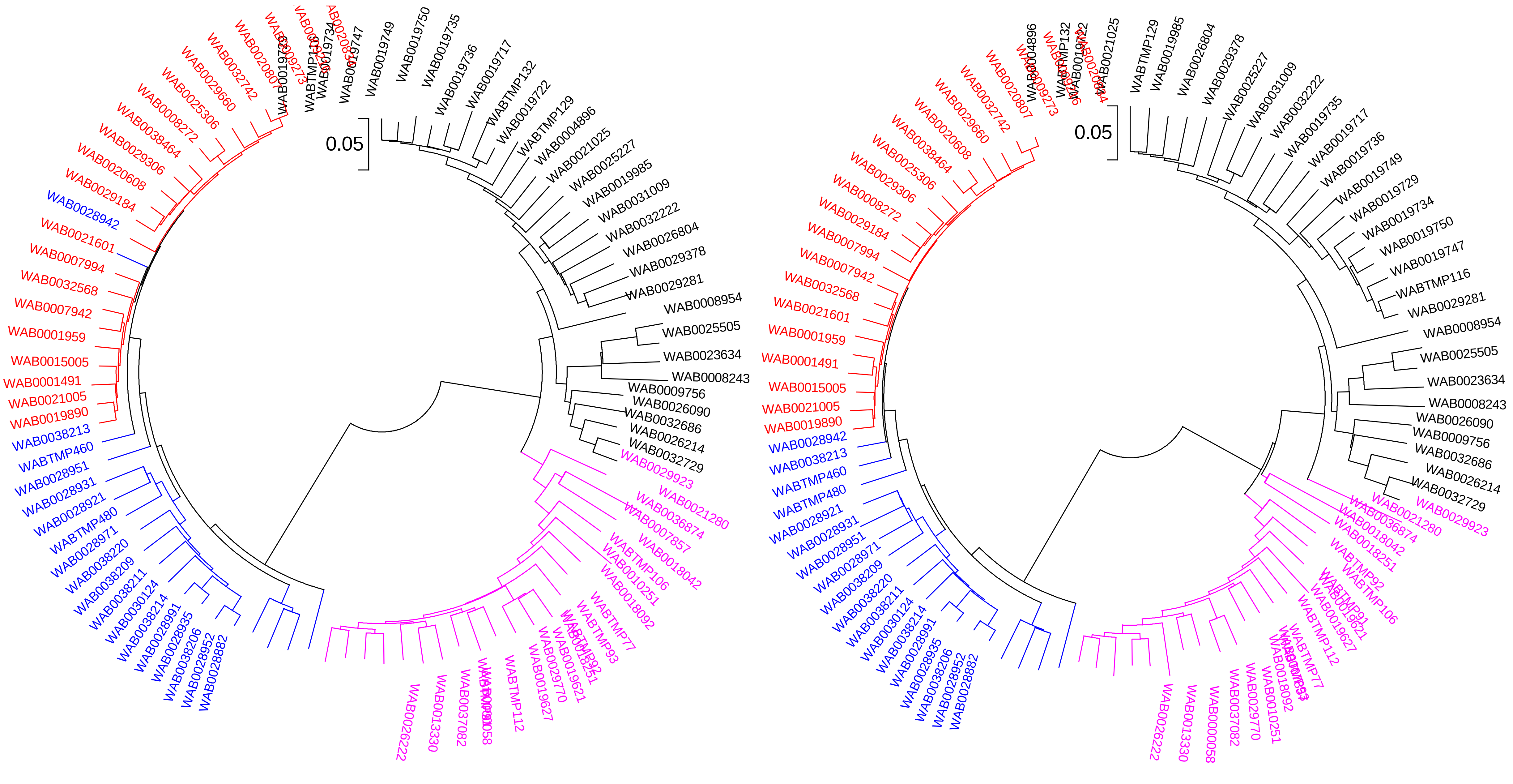

(g) Set-7

(h) Set-8

Supplementary Fig. S7 (Page 4 of 6)

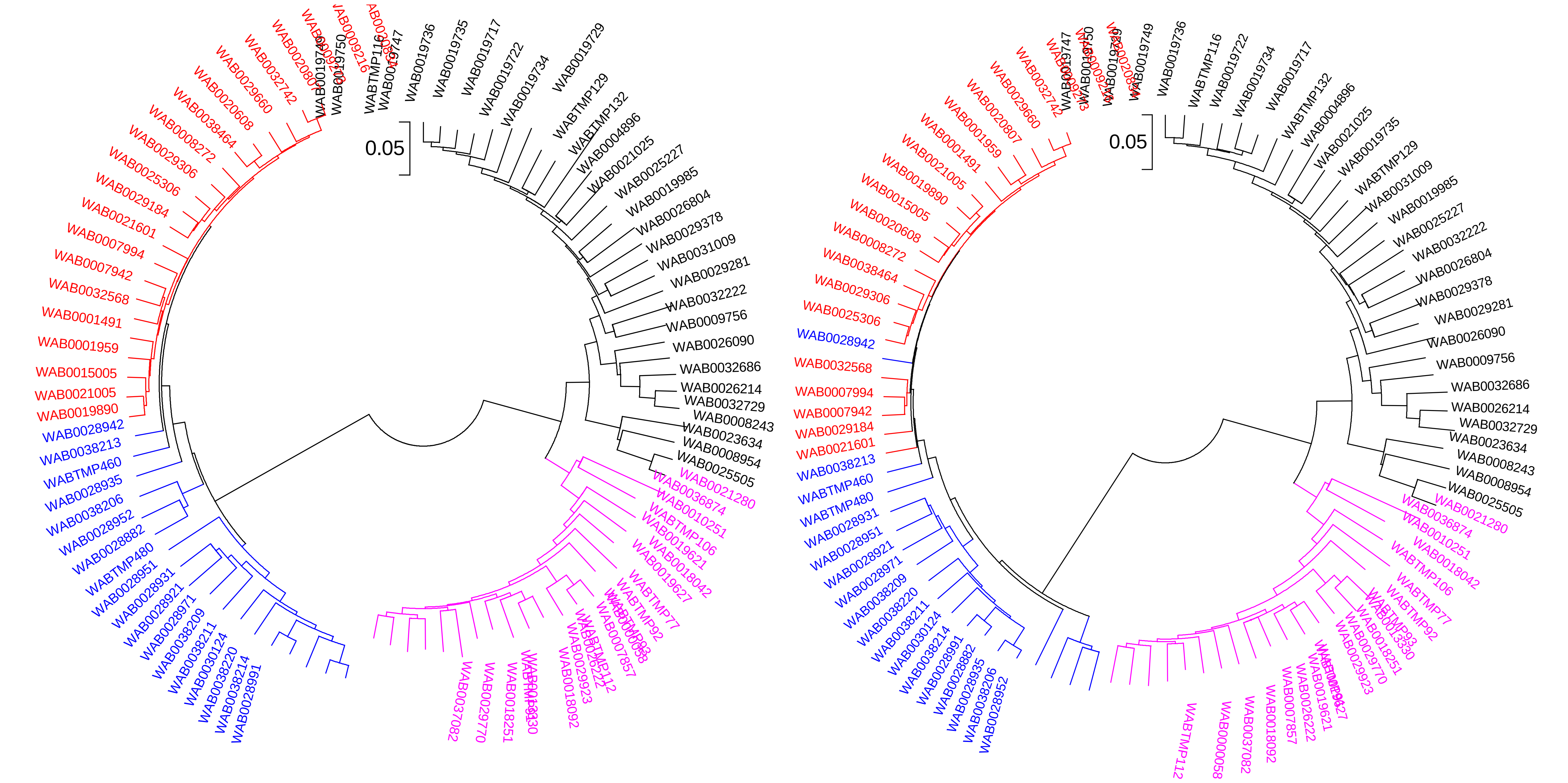

(i) Set-9

Supplementary Fig. S7 (Page 5 of 6)

(j) Set-10

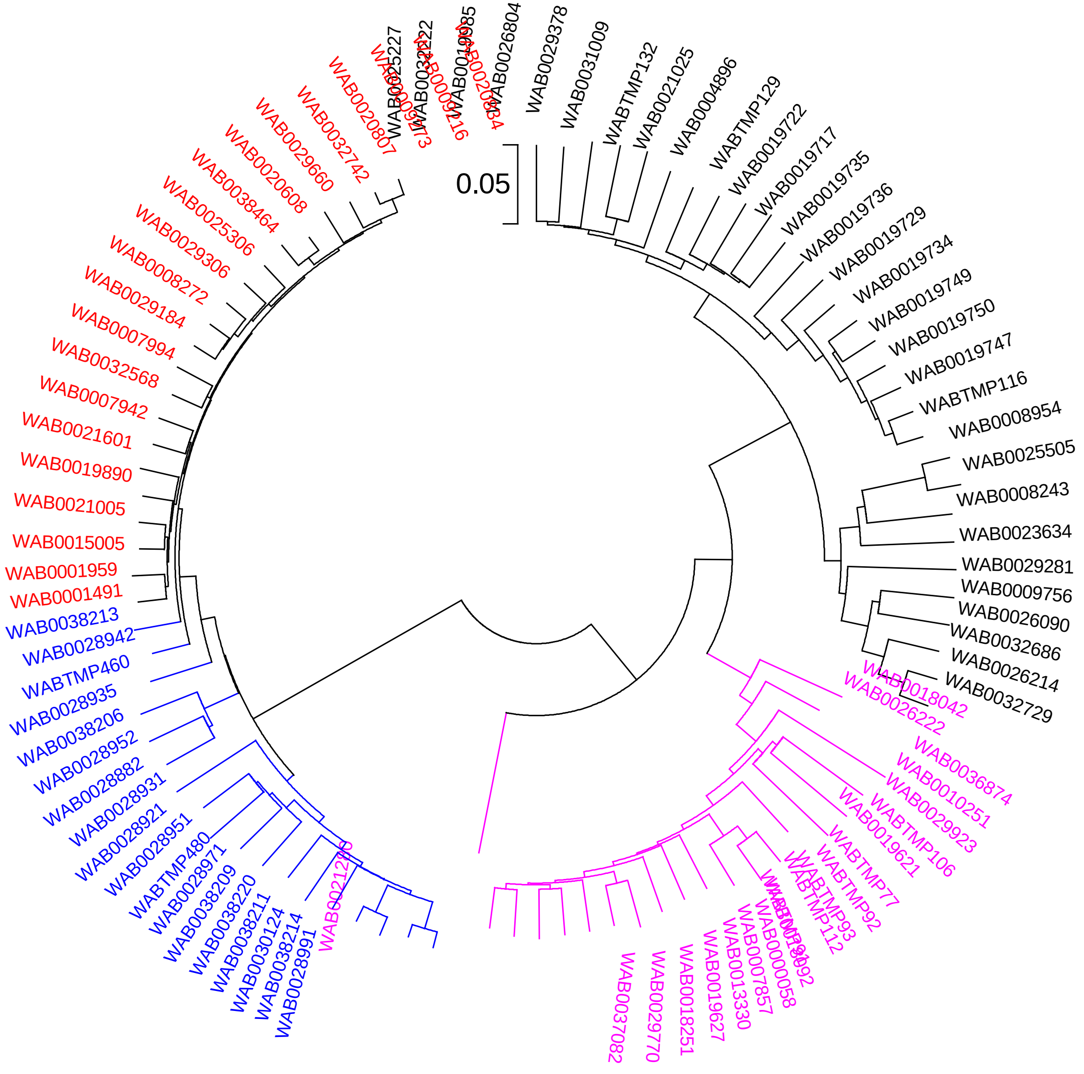

(k) Set-11

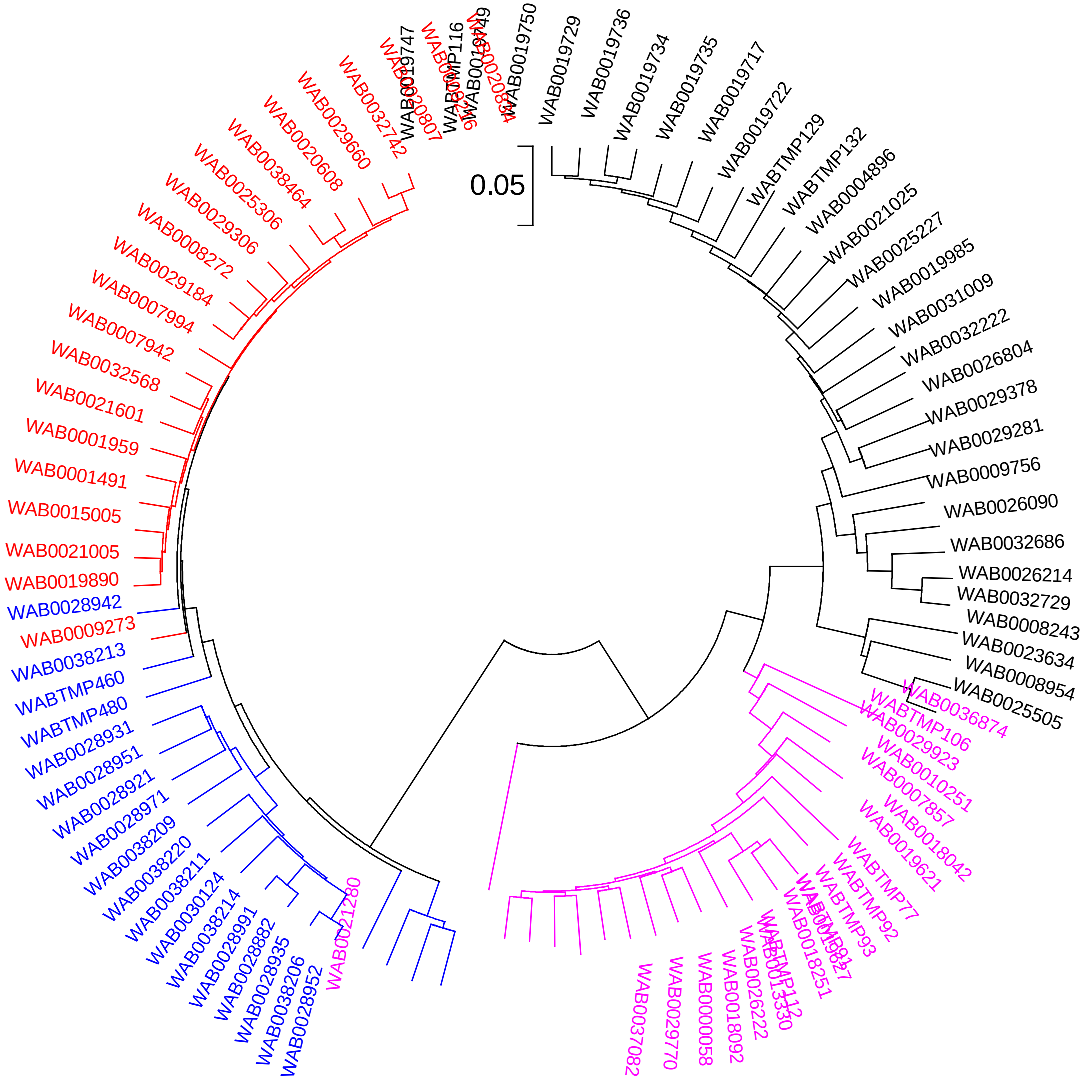

(L) Set-12

Supplementary Fig. S7 (Page 6 of 6)

Supplementary Fig. S8 Plots of PC1 and PC2 from principal component analyses of 90 accessions based on 46,818 SNPs. Each accession was represented by (a) 17 DNA samples consisting of 15 individuals plus two bulks of 5 plants and 10 plants (Set-1); (b) bulks of 5 plants (Set-3); (c) bulks of 10 pants (Set-4); (d-i) a randomly selected individual out of Set-2, which is repeated 6-times (Set-5 to Set-10); (j) an average of 5 individuals (Set-11); and (k) an average of 10 individuals (Set-12). Both Set-11 and Set-12 form Set-2. Accessions are colored depending on their species and/or group: *O. glaberrima* (red), *O. barthii* (blue), *O. sativa* adapted to the upland ecology (pink) and lowland ecology (black).

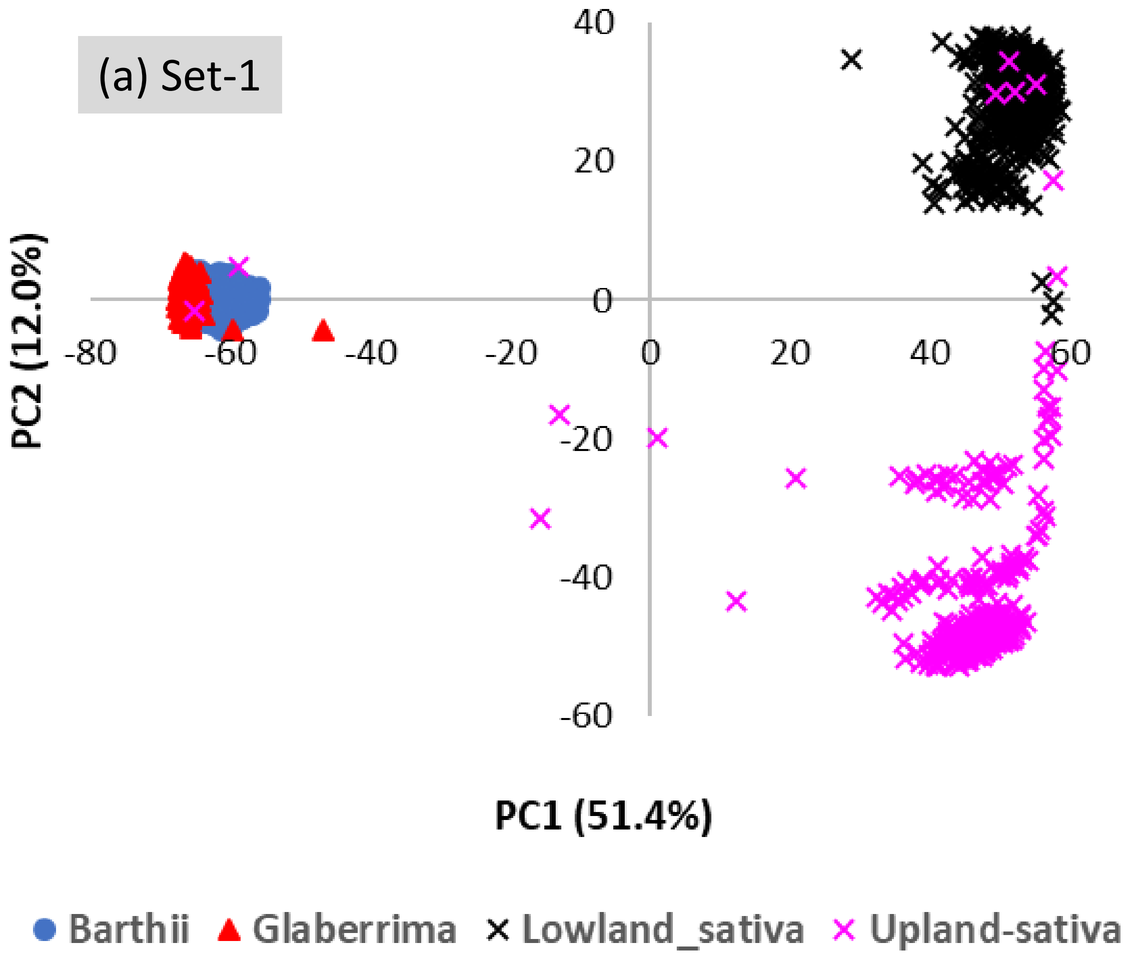

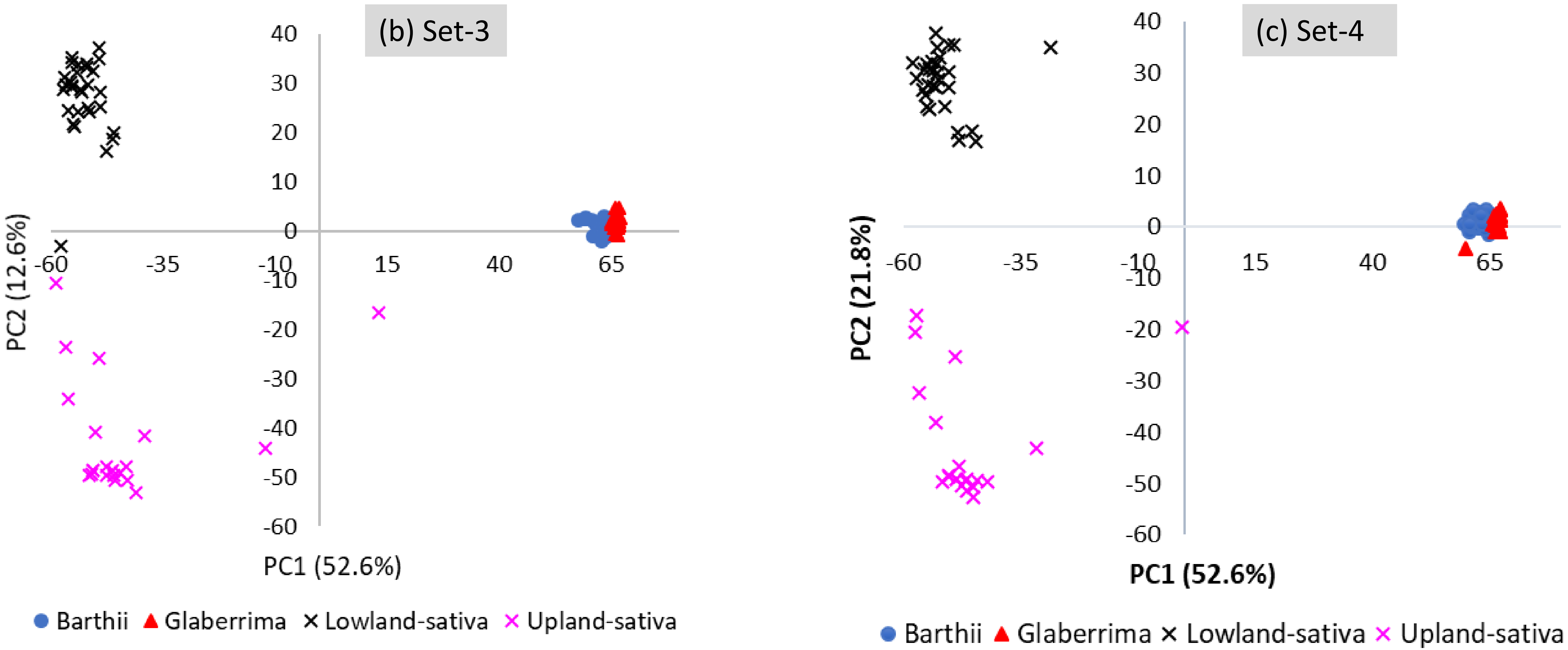

Supplementary Fig. S8 (Page 2 of 6)

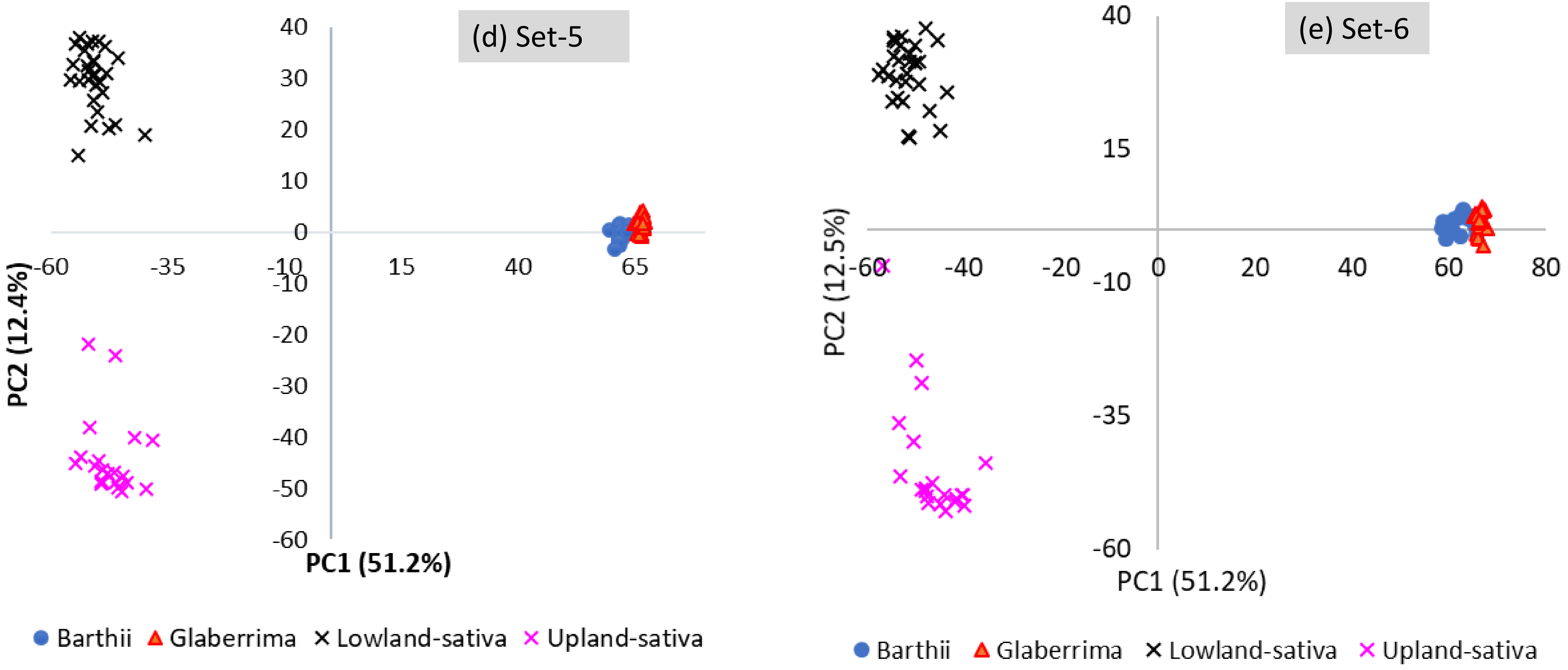
(d) Set-5
40
30
20
10
0
-10
-20
-30
-40
-50
-60
-60
-35
-10
15
40
65
PC1 (51.2%)
PC2 (12.4%)
Barthii
Glaberrima
Lowland-sativa
Upland-sativa
(e) Set-6
40
15
-10
-35
-60
-60
-40
-20
0
20
40
60
80
PC1 (51.2%)
PC2 (12.5%)
Barthii
Glaberrima
Lowland-sativa
Upland-sativa

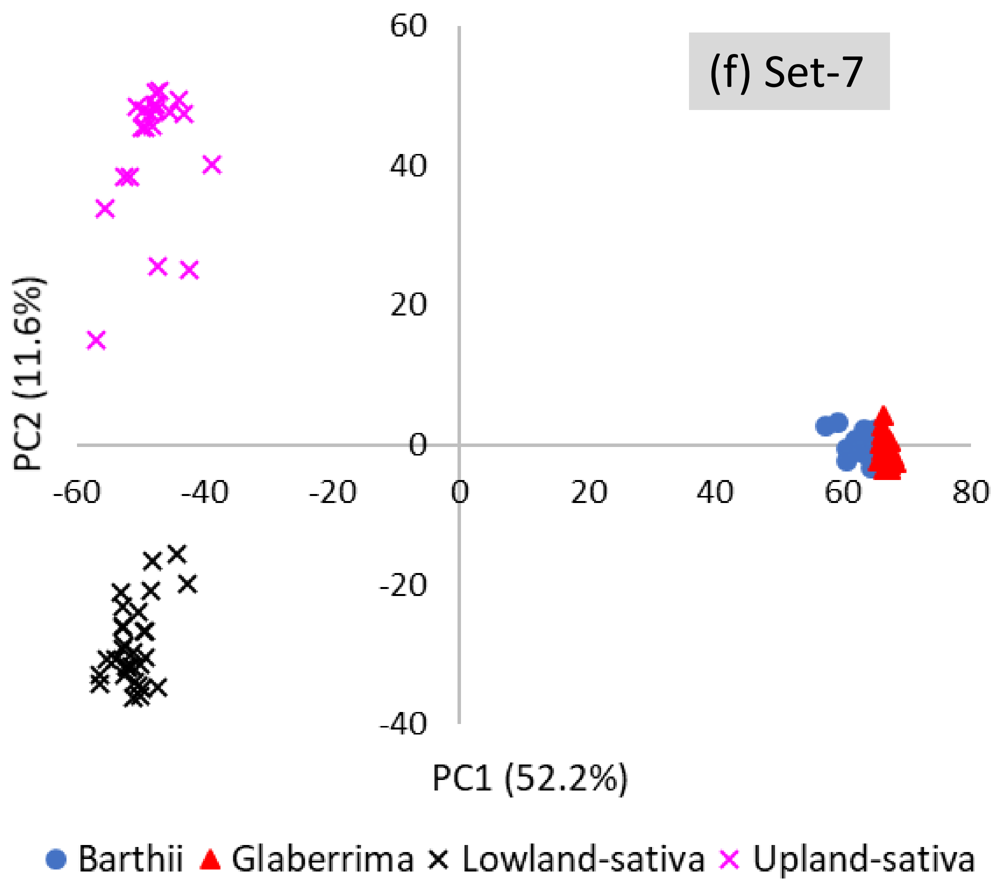

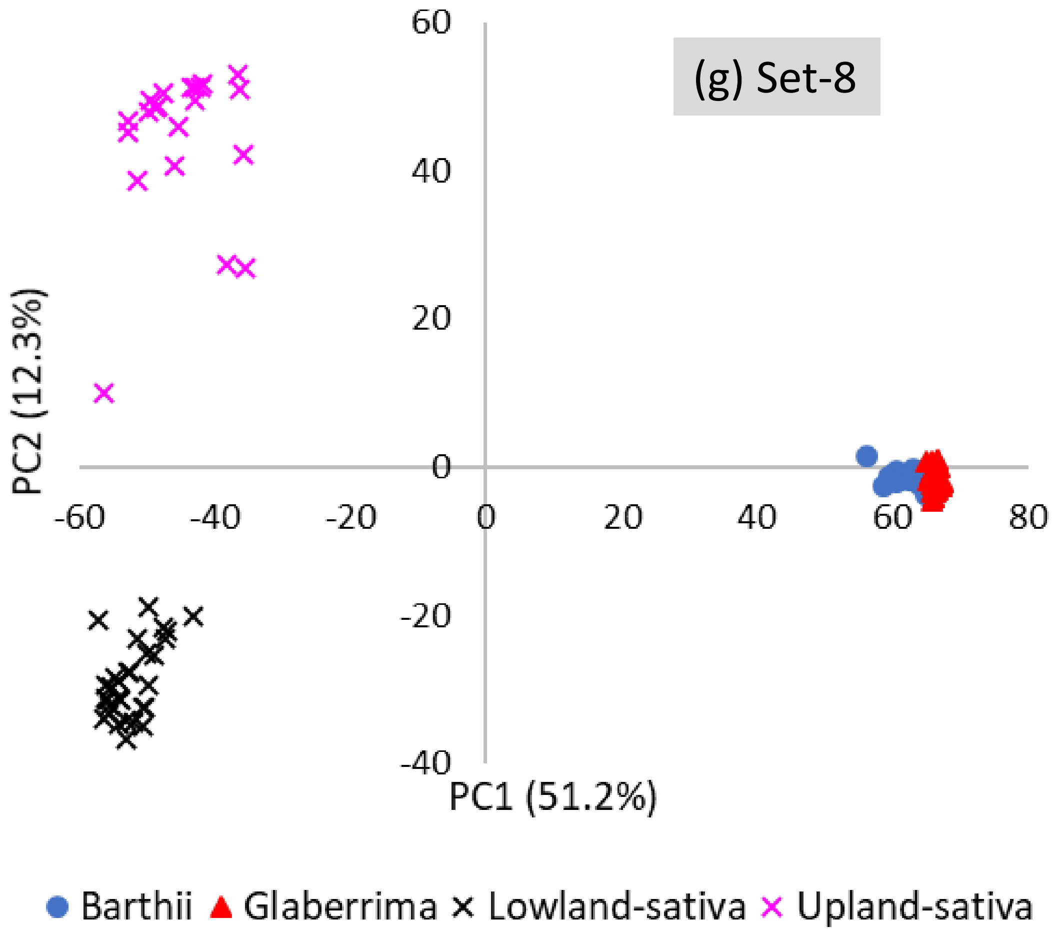

Supplementary Fig. S8 (Page 4 of 6)

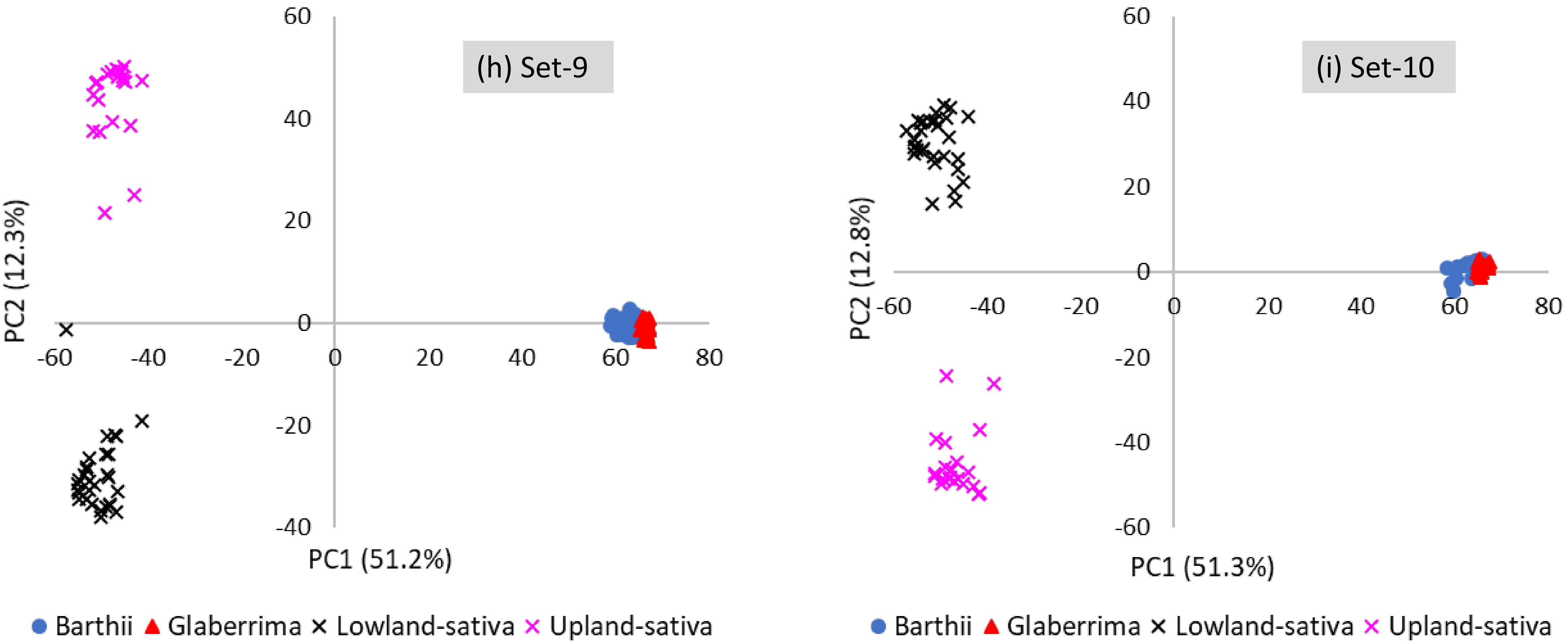

Supplementary Fig. S8 (Page 5 of 6)

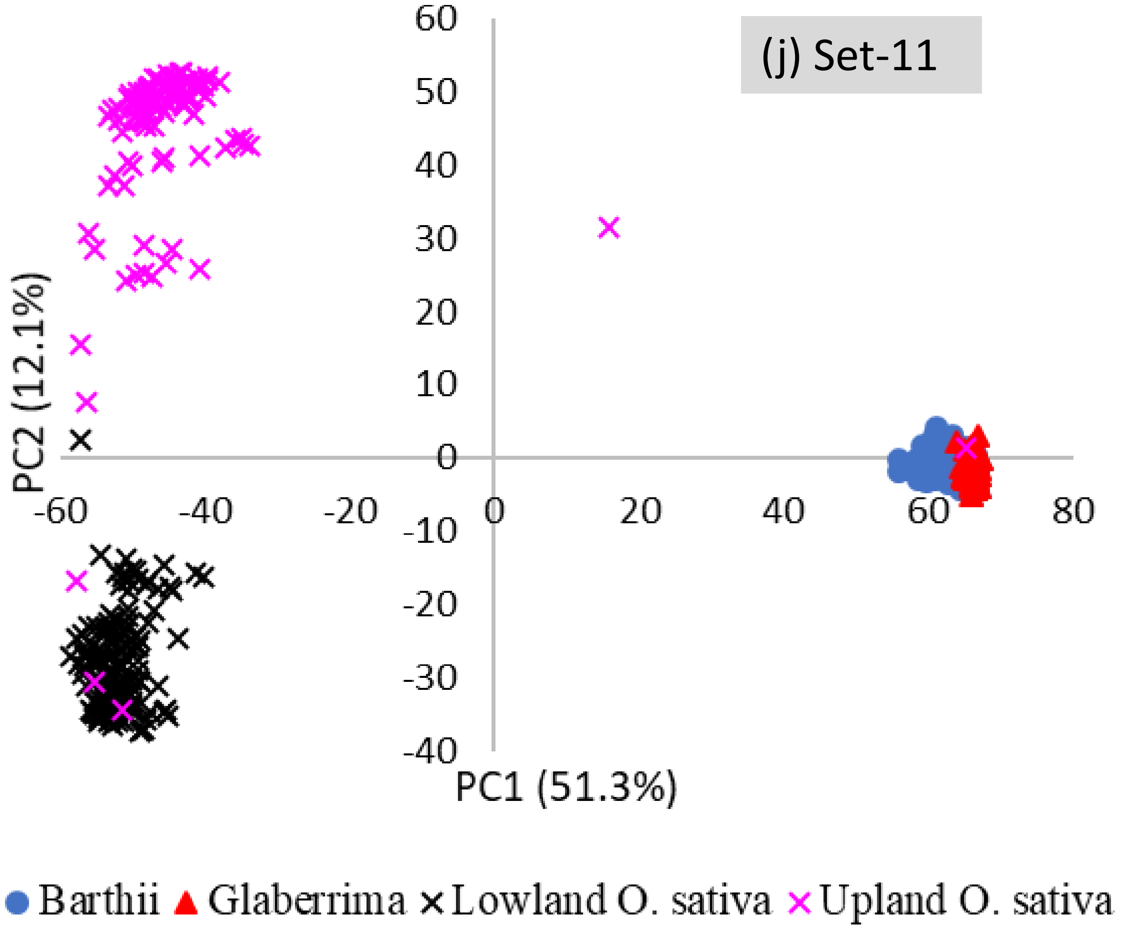

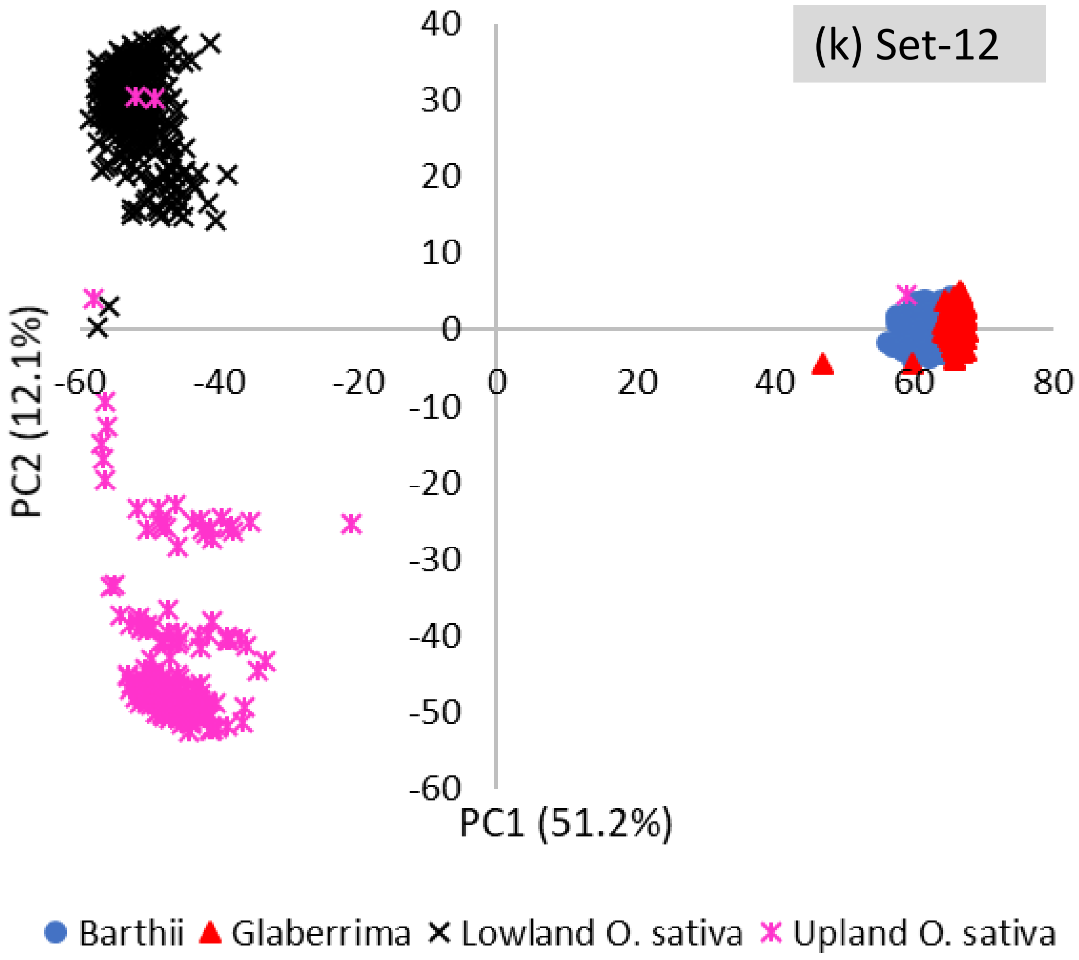

Supplementary Fig. S8 (Page 6 of 6)

Supplementary Fig. S9 Summary of the partitioning of the molecular variation (AMOVA) of 90 accessions, each represented by 9 datasets corresponding to 15 individuals (Set-2), bulks of 5 plants (Set-3), bulks of 10 plants (Set-4) and randomly selected single plants out of Set-2 (Set-5 to Set-10). The five groups include *O. glaberrima*, *O. barthii*, *O. sativa* spp. indica, *O. sativa* spp. japonica, and interspecific improved genotypes. In the case of four groups, *O. sativa* and interspecific genotypes were assigned into lowland *O. sativa* and upland *O. sativa* based on phylogenetic and population structure analyses. The three groups are the same as the four groups except that *O. barthii* and *O. glaberrima* were merged into the same group. See Supplementary Table S11 for details.

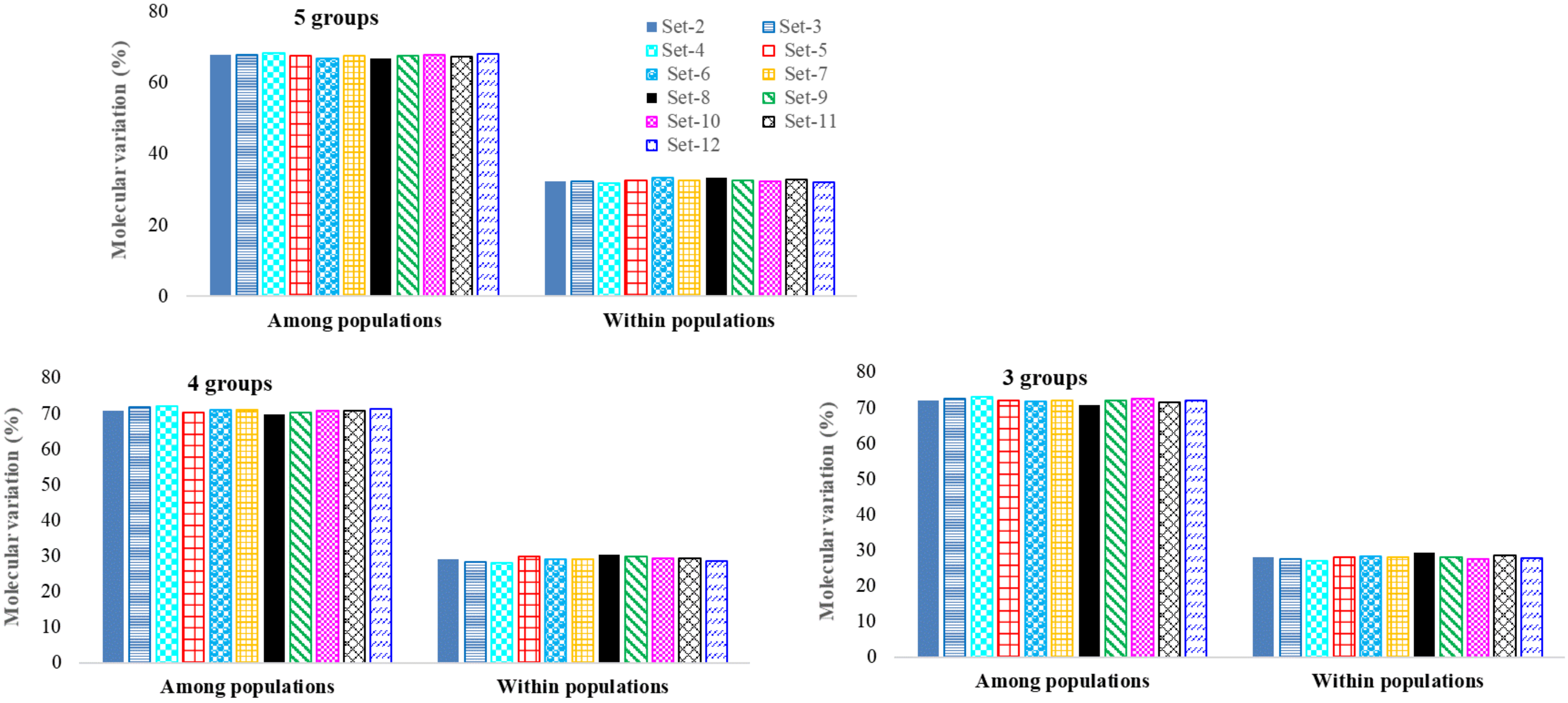

Supplement: Supplementary file 2 — Supplementary Table S1 [file 41598_2020_70842_MOESM2_ESM.pdf]
